# Cooperative antibiotic resistance facilitates horizontal gene transfer

**DOI:** 10.1038/s41396-023-01393-1

**Published:** 2023-03-22

**Authors:** Qinqin Wang, Shaodong Wei, Ana Filipa Silva, Jonas Stenløkke Madsen

**Affiliations:** 1 Department of Biology, University of Copenhagen, 2100 Copenhagen, Denmark; 2 National Food Institute, Technical University of Denmark, 2800 Lyngby, Denmark

**Keywords:** Bacterial evolution, Antibiotics

## Abstract

The rise of β-lactam resistance among pathogenic bacteria, due to the horizontal transfer of plasmid-encoded β-lactamases, is a current global health crisis. Importantly, β-lactam hydrolyzation by β-lactamases, not only protects the producing cells but also sensitive neighboring cells cooperatively. Yet, how such cooperative traits affect plasmid transmission and maintenance is currently poorly understood. Here we experimentally show that KPC-2 β-lactamase expression and extracellular activity were higher when encoded on plasmids compared with the chromosome, resulting in the elevated rescue of sensitive non-producers. This facilitated efficient plasmid transfer to the rescued non-producers and expanded the potential plasmid recipient pool and the probability of plasmid transfer to new genotypes. Social conversion of non-producers by conjugation was efficient yet not absolute. Non-cooperative plasmids, not encoding KPC-2, were moderately more competitive than cooperative plasmids when β-lactam antibiotics were absent. However, in the presence of a β-lactam antibiotic, strains with non-cooperative plasmids were efficiently outcompeted. Moreover, plasmid-free non-producers were more competitive than non-producers imposed with the metabolic burden of a plasmid. Our results suggest that cooperative antibiotic resistance especially promotes the fitness of replicons that transfer horizontally such as conjugative plasmids.

## Introduction

Cooperation between microorganisms is often facilitated by the production and secretion of extracellular products, which function as a group resource that persists in the environment and serves as public goods [1], such as β-lactamases [2], siderophores [3], and quorum-sensing autoinducers [4]. Cooperators that produce public goods generally bear a metabolic burden associated with synthesizing and releasing the public goods, while neighboring non-producers, also termed non-cooperators, can benefit from the public goods produced by cooperators, yet do not pay the cost to produce them. Cooperation is therefore vulnerable against non-cooperators as they can outcompete producers, which can lead to the downfall of the cooperative phenotype. This is known as the public goods dilemma [5].

Although the public goods dilemma represents a significant barrier to cooperative behaviors, cooperation is promoted and maintained through diverse properties and phenotypes that safeguard the costly public goods. This, for example, is done by facilitating selection towards kin in accordance with Hamilton’s theories on inclusive fitness [6]. An example is growth in biofilms, where the matrix provides structure, which increases the genetic relatedness of neighbors. Another example is metabolic prudence, here public goods are regulated and only secreted when the cost of their production and impact on individual fitness is low.

Horizontal gene transfer (HGT) has been proposed to act as a mechanism that can stabilize public goods cooperation by converting non-cooperative cells into cooperators [7, 8]. This is an appealing postulate because HGT is rampant among bacteria, especially when mediated by mobile genetic elements such as conjugative plasmids. Plasmids are DNA elements that replicate extra-chromosomally within bacterial genomes. They typically transfer horizontally by means of conjugation but can also do so by transformation, transduction, or through extracellular vesicles. Plasmids are infamous for spreading genes involved in virulence [9], ecological interaction [10], and are the main mechanism for the spread of antimicrobial resistance (AMR) [11–13]. Moreover, a prevalence of predicted public goods genes has been shown to be carried by plasmids of several different bacterial taxa, for example, the *Enterobacteriaceae* family that includes *Escherichia coli* [14].

AMR is a rising global and critical public health concern [15] and up to 10 million yearly deaths have been predicted to be associated with AMR by 2050 [16]. Especially a growing number of carbapenemase-producing *Enterobacteriaceae* cause increased treatment times and mortality rates [17]. Carbapenems are one of the most potent β-lactams for treating bacterial infections, especially infections with *Enterobacteriaceae* that produce extended-spectrum β-lactamases [18], and carbapenem-resistant bacteria are thus a critical problem [19]. The escalating global antibiotic resistance crisis is largely due to the spread of plasmid-borne antibiotic resistance genes [20] including β-lactamases [21]. Furthermore, β-lactamases have been shown to be public good antibiotic resistances that do not only protect the producer, but also the neighboring susceptible bacteria [2, 22, 23]. Yet, the interconnection between β-lactamases as public goods and their transfer by conjugative plasmids have not been disclosed. Here, we studied this interconnection using KPC-2 (*Klebsiella pneumoniae* carbapenemase-2), a carbapenemase that spreads horizontally through mobile genetic elements and mainly *via* plasmids [24–26]. KPC β-lactamases are the main cause of β-lactam resistance among bacteria belonging to *Enterobacteriaceae*, which not only act against carbapenems but also cephalosporins [27]. Thus, the horizontal spread of *bla*_KPC_ genes is an important cause of multidrug-resistant Gram-negative pathogens [28, 29].

The interconnection between HGT and public goods has previously been investigated by researchers mainly by computational approaches [7, 30–32], and a smaller number of in vitro studies have been done, for example, the work by Dimitriu et al. [31], where the quorum-sensing autoinducer C4-HSL was used as a model public good to investigate the role of HGT in promoting cooperation. In contrast, here we specifically studied how wild-type public goods production, in the form of antibiotic resistance, affects transmission and maintenance of conjugative plasmids in vitro in mixed bacterial populations.

## Results

### An experimental model of cooperative antibiotic resistance

We first designed an experimental model based on *E. coli*. Our assumption was that the KPC-2 β-lactamase would act as a public good in the presence of the β-lactam imipenem. *E. coli* MG1655 was complemented with *sfGFP* (green fluorescence) and *bla*_KPC-2_ expressed from its wild-type promoter, either in (i) the chromosome; (ii) a conjugative plasmid (pKJK5); or (iii) a non-conjugative mutant of the plasmid (pKJK5_NC_). This way three distinct β-lactamase producers were constructed, referred to as the chromosomal producer (***P***_***chr***_), the conjugative plasmid producer (***P***_***conj***_), and the non-conjugative plasmid producer (***P***_***non-conj***_). An otherwise isogenic non-producer *E. coli* MG1655 (***N***) tagged with *mCherry* (red fluorescence) was also constructed. In experiments with conjugative plasmids, we distinguish between the original non-producer population (***N***), which may also include transconjugants (***T***), and the remaining non-producer population (***N***_***rem***_) which are non-producers that have not acquired the plasmid. The minimal inhibitory concentrations (MIC) of imipenem and tetracycline were tested for all strains (Table S1) and their growth was compared (Fig. S1, *n* = 6). Co-cultures of a producer (either ***P***_***chr***_, ***P***_***conj***_, or ***P***_***non-conj***_) and ***N*** were grown as colonies on agar medium for up to six days with or without antibiotics (120% above-MIC levels of ***N***). The different populations in the communities were monitored by flow cytometry and fluorescent stereo microscopy (Fig. S2). This setup was chosen as it previously has been shown that growing co-cultures of β-lactamase producers and non-producers in colonies on agar plates favor the rescue of non-producers when compared with growth in broth media [23, 33].

### Plasmid-encoded KPC-2 elevates both intracellular and extracellular β-lactamase activity and enhances the rescue of non-producers

Plasmids are often present in their bacterial hosts in multiple copies and the wild-type plasmid pKJK5 used here has a copy number of 5–7 per cell [34, 35]. Plasmids that encode AMRs may, as an effect of higher copy numbers, increase the MIC of their host compared with the same AMR encoded as a single copy in the chromosome [36, 37]. Since higher expression of plasmid-encoded AMRs is a strongly selected trait during bacterial AMR evolution [38], we hypothesized that the multi-copy nature of plasmids would promote the protective effect of β-lactamase producers on non-producers as more β-lactamases are expressed to hydrolyze extracellular antibiotic.

**Fig. 1:**
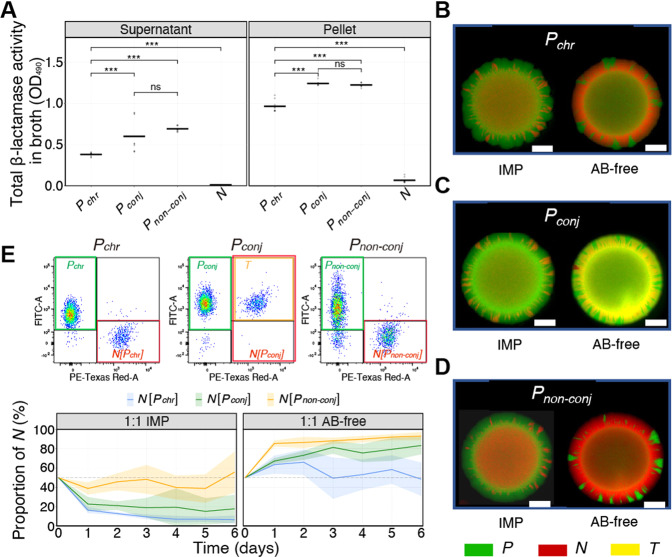
Rescue is enhanced when cooperative antibiotic resistance is expressed from plasmids. **A** Total intracellular and extracellular β-lactamase activity of strains ***P***_***chr***_ (*bla*_KPC-2_ encoded in chromosome), ***P***_***conj***_ (*bla*_KPC-2_ encoded in conjugative plasmid), ***P***_***non-conj***_ (*bla*_KPC-2_ encoded in non-conjugative plasmid), and ***N*** (non-producer) in broth cultures. The dots and lines refer to the data points (*n* = 9) and means, respectively. *p*_adj_ values were derived from one-way ANOVA followed by a post-hoc Tukey test: *: 0.01 < *p*_adj_ < 0.05; **: 0.001 < *p*_adj_ < 0.01; ***: *p*_adj_ < 0.001. **B**–**D** Representative fluorescence microscopy images of colonies illustrating producers (***P***, green), non-producers (***N***, red) and transconjugants (***T***, yellow) after one day of co-cultivation. White scale bars represent 5 mm. **E** Top: schematic diagram showing the use of flow cytometry to distinguish producers (green box) and non-producers (red box) for the first day co-cultures with imipenem (IMP). Bottom: proportion of the original non-producer populations (***N***) rescued in co-cultures with the three different KPC-2 producers ***N***[***P***_***chr***_], ***N***[***P***_***conj***_] or ***N***[***P***_***non-conj***_], in the presence of IMP and without antibiotics (AB-free), over six days. Co-cultures were initiated at a 1:1 ratio of producers and non-producers. In ***P***_***conj***_ co-cultures, ***N*** is the sum of the transconjugants (***T***) and plasmid-free non-producers (***N***_***rem***_). Lines show the mean of six replicates (*n* = 6) and shaded areas are 95% confidence intervals of the mean.

We quantified the total β-lactamase activity of the different strains in broth cultures (Fig. 1A, *n* = 9). The plasmid-carrying strains (***P***_***conj***_ and ***P***_***non-conj***_) had similar but higher β-lactamase activities than ***P***_***chr***_ and ***N***, both extracellularly and intracellularly (adjusted *p* value (*p*_adj_) < 0.001, Table S2). The intracellular β-lactamase activities of all producers were higher than that of the extracellular activities (*p*_adj_ < 0.001, Table S2). Next, we evaluated how copy number (plasmid *vs*. chromosome) and β-lactamase activity affects the putative cooperative sharing of β-lactam resistance, by quantifying non-producer survival in co-cultures with producers in the presence of imipenem. Producers (***P***_***chr***_, ***P***_***conj***_, or ***P***_*non-conj*_) and non-producers (***N***) were co-cultured at an initial ratio of 1:1 and followed for six days, either with or without imipenem (0.6 µg/ml IMP or AB-free) (Fig. S3).

Looking at the fluorescence patterns of the colony images (Fig. 1B–D, Fig. S3) it was seen that distinct populations radiate outwards, over time, from the central mixing zone where the droplets initially were placed on the agar medium. In experiments without the conjugative plasmids, the radiating patches consisted of specific populations (green producers or red non-producers) suggesting that intermixing was minor. In contrast, in experiments with ***P***_***conj***_ radiating patches with cells that were both green and red (depicted as yellow) were observed, implying that most non-producers had become transconjugants. Furthermore, the colony images revealed that the width of the radially expanding ***N*** populations in co-cultures with ***P***_***non-conj***_ was wider than in those with ***P***_***chr***_. This supports a higher expression of β-lactamases by ***P***_***non-conj***_ compared with ***P***_***chr***_, and long-range cooperative resistance at a global scale [39].

Cells were also enumerated by flow cytometry (Fig. 1E and Fig. S4A, *n* = 6) which showed that all β-lactamase producers protected the non-producers in the presence of above-MIC levels of IMP. This demonstrated that KPC-2 is a public good, though the extent of protection was different. After one day of co-cultivation in the presence of IMP, the average relative proportion of ***N*** in ***P***_***non-conj***_ co-cultures was higher (36.1%) than those in ***P***_***chr***_ (16.4%, *p*_adj_ < 0.001, Table S2) and ***N*** in ***P***_***conj***_ co-cultures (20.5%, *p*_adj_ < 0.001, Table S2). During the following five days, the differences in the relative proportion of ***N*** across groups remained similar (*p*_adj_ < 0.001, pairwise comparison, Tabel S2). The β-lactamase activity of ***P***_***chr***_ and ***P***_***non-conj***_ and the relative proportion of protected ***N*** (*ρ* = 0.87, *p* < 0.001), respectively, was positively correlated. In parallel experiments without imipenem (AB-free), the relative proportion of ***N*** in ***P***_***non-conj***_ co-cultures was higher than those in ***P***_***chr***_ co-cultures (*p*_adj_ < 0.001, Table S2). This implies that it was more costly to maintain the plasmid and express the β-lactamase from plasmid replicons compared with the chromosome. Moreover, the proportion of ***N*** stayed closer to 50% in ***P***_***chr***_ co-cultures (on average 56.5% over six days), suggesting that the fitness of ***P***_***chr***_ was more similar to ***N***, and a relatively low cost of chromosomal *bla*_KPC-2_ expression.

HGT may facilitate the stable acquisition of cooperative genes [40], but plasmid carriage can also impose a fitness burden on the host. Hence, the effect of plasmid acquisition in ***P***_***conj***_ co-cultures was investigated. The flow cytometry data (Fig. 1E and Fig. S4A) showed that in the ***P***_***conj***_ co-cultures, most of the ***N*** population was noticeably efficiently converted into transconjugants. This supported that the yellow radiating patches of the colony images of ***P***_***conj***_ co-cultures (Fig. 1B–E, Fig. S3) were indeed cells that are fluorescing both green and red. In the absence of IMP (AB-free), ***N*** made up a smaller proportion when co-cultured with ***P***_***conj***_ compared with ***P***_***non-conj***_ (Fig. 1E). This effect was also observed when IMP was present (Fig. 1E). ***P***_***non-conj***_ and ***P***_***conj***_ had similar β-lactamase activities, both extracellular and intracellular (Fig. 1A, *p*_adj_ > 0.05, Table S2), but the proportion of ***N*** was lower in ***P***_***conj***_ co-cultures because conjugation converted part of the ***N*** population into producers (***T***).

### Public good resistance increases the probability of plasmid transfer to distinct genotypes

**Fig. 2:**
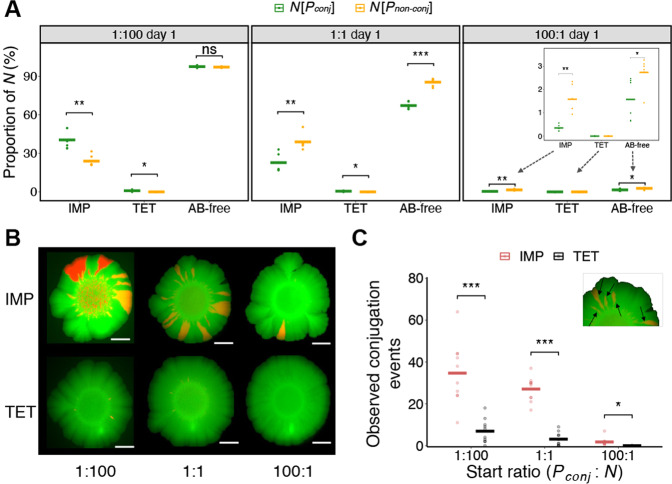
Efficient social conversion by conjugation of cooperative antibiotic resistance. **A** Proportion of the original non-producer populations (***N***) after one day of co-cultivation with either producer ***P***_***conj***_ (*bla*_KPC-2_ encoded in conjugative plasmid) or ***P***_***non-conj***_ (*bla*_KPC-2_ encoded in non-conjugative plasmid). ***P***_***conj***_ or ***P***_***non-conj***_ were mixed with ***N*** at different start ratios (1:100, 1:1 and 100:1) and co-cultured in the presence of imipenem (IMP), tetracycline (TET), or without antibiotics (AB-free). Here ***N*** refers to the original non-producer populations rescued in co-cultures and thus includes transconjugants (***T***) in the case of ***P***_***conj***_ co-cultures. Dots represent individual data points (*n* = 6) and lines are the mean. **B** Representative fluorescence microscopy images of colonies illustrating producers (green), non-producers (red) and transconjugants (yellow) after four days of co-cultivation. White scale bars represent 10 mm. **C** The number of observed conjugation events was calculated based on stereo microscopy images. Each black arrow represents an individual transfer event. Dots represent individual data points (*n* = 10) and lines are mean. For (**A**) and (**C**), *p*_adj_ values were derived from two-sided *t*-test, *: 0.01 < *p*_adj_ < 0.05; **: 0.001 < *p*_adj_ < 0.01; ***: *p*_adj_ < 0.001.

We compared conjugation when exposed to antibiotics that are evaded by either public good resistance *vs*. private good resistance. Besides *bla*_KPC-2_, pKJK5 and pKJK5_NC_ also encode resistance towards tetracycline which, is mediated by an efflux pump [41] and can thus be considered a private good resistance, as it solely protects the producer cells. Co-culture experiments with ***N*** and ***P***_***non-conj***_ or ***P***_***conj***_ were performed in the presence of IMP, tetracycline (TET, 7 µg/ml), or without antibiotics (AB-free). Besides experiments initiated at producer to non-producer ratios of 1:1, experiments with initial ratios of 1:100 and 100:1 were also conducted (Fig. 2A, Fig. S3 and Fig. S5–7, *n* = 6). In the presence of IMP we observed that ***N*** survived at all initial ratios and the proportions of ***N*** were significantly higher than zero (*p*_adj_ < 0.001, one-sample one-sided t-test, Table S2), confirming the protective effect of KPC-2. Relative to co-cultures with ***P***_***non-conj***_, the proportion of surviving ***N*** (including both ***N***_***rem***_ and ***T***) in ***P***_***conj***_ co-cultures was lower when the initial ratio of producer to non-producer was 1:1 and 100:1, both with and without IMP. In AB-free co-cultures part of the ***N*** population were ***T***, which were less fit than ***N***_***rem***_ as also seen for ***N*** in ***P***_***non-conj***_ co-cultures. ***N*** were relatively frequent in ***P***_***non-conj***_ co-cultures when IMP was present, but in ***P***_***conj***_ co-cultures conjugation efficiently converted the ***N*** population into producing transconjugants (***T***) (Fig. S8, *n* = 6). Moreover, when the initial ratio of producers was low (1:100 ratio) and IMP was present, fewer ***N*** received the plasmid leading to a similar proportion of ***N*** in both co-cultures with ***P***_***conj***_ and ***P***_***non-conj***_ (Fig. 2A, AB-free). Conversely, at the 1:100 ratio, the proportion of ***N*** in IMP co-cultures with ***P***_***conj***_ was higher than in ***P***_***non-conj***_ (Fig. 2A, IMP).

Unlike co-cultures with IMP, when treated with TET, ***N*** did not grow in any of the ***P***_***non-conj***_ co-cultures (Fig. 2A and Fig. S8A) showing that the tetracycline resistance was indeed a private good. In ***P***_***conj***_ co-cultures with TET, no ***N*** was detected when the initial producer ratio was high (100:1). Only a few ***N*** grew at ratios 1:100 and 1:1 and these were all transconjugants (Fig. S8B). We noted that successful transconjugant proliferation post conjugation (days two to six) was very rare. Colony images further demonstrate that no ***N*** were observed when selecting with TET and very few ***T*** were detected (Fig. 2B and Fig. S7).

In communities with many different genotypes, the success of a plasmid may increase with the number of genotypes it associates with – essentially by “hedging its bets”. To access the potential of conjugation to unique genotypes under the selection of IMP and TET, we enumerated individual transfer events based on the colony images acquired by fluorescent microscopy (Fig. 2C, *n* = 10) [42]. Each transconjugant lineage that radiated out as a patch (depicted as yellow) in the colonies was considered a hypothetical unique genotype that originated from a single parent cell. By enumerating these manually, it was found that a higher number of distinct genotypes received the plasmid when few donors/producers were present (1:100 or 1:1), compared with when many were present (100:1). Also, more unique genotypes were converted when treated with IMP compared with TET (Fig. 2B). These results support that public good resistance provides more potential recipients for conjugal transfer while also facilitating social conversion of non-cooperators.

### Efficient social conversion by plasmid conjugation

**Fig. 3:**
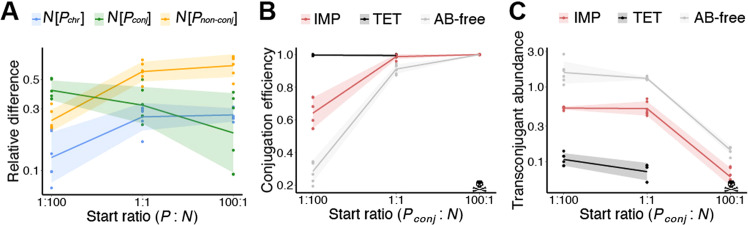
A distinction between conjugation efficiency and transconjugant abundance for conjugative plasmids when selecting for private vs. public antibiotic resistance. **A** Relative differences (IMP/AB-free) in abundances of non-producer populations (***N***) when cultivated with the different producers [***P***_***chr***_], [***P***_***conj***_] and [***P***_***non-conj***_] in the presence of imipenem (IMP) and without antibiotics (AB-free), started at different initial producer to non-producer ratios (100:1, 1:1, and 1:100). **B** Conjugation efficiencies ($\frac{{{{{{{{\boldsymbol{T}}}}}}}}}{{{{{{{{{\boldsymbol{T}}}}}}}} + {{{{{\boldsymbol{N}}}}}}_{{{{{{{\mathbf{{rem}}}}}}}}}[{{{{{\boldsymbol{P}}}}}}_{{{{{{{\mathbf{{conj}}}}}}}}}]}}$), calculated for *P*_*conj*_ co-cultures exposed to imipenem (IMP), tetracycline (TET), or without antibiotics (AB-free). **C** Transconjugant abundances ($\frac{{{{{{{{\boldsymbol{T}}}}}}}}}{{\sqrt {{{{{{{{\boldsymbol{P}}}}}}_{{{{{{{\mathbf{conj}}}}}}}}}} \times \left( {{{{{{{{\boldsymbol{T}}}}}}}} + {{{{{{{\boldsymbol{N}}}}}}_{{{{{{{\mathbf{rem}}}}}}}}}}\left[ {{{{{{{{\boldsymbol{P}}}}}}_{{{{{{{\mathbf{conj}}}}}}}}}}} \right]} \right)} }}$), calculated for ***P***_***conj***_ co-cultures exposed to IMP, TET, or AB-free. For (**A**), (**B**), and (**C**), dots represent individual data points (*n* = 6), and lines connect the mean for co-cultures started at different initial producer to non-producer ratios and incubated for 24 h. Shaded areas are 95% confidence intervals of the mean. The skull and crossbones symbols indicate that no transconjugants were identified in 100:1 samples exposed to TET.

To delineate the dynamics of social conversion by plasmid conjugation, we compared the relative difference between ***N*** abundances in co-cultures with IMP and those without **(**Fig. 3A, *n* = 6) after one day of cultivation. In co-cultures where conjugation could not occur (***P***_***chr***_ and ***P***_***non-conj***_), the relative difference of ***N*** abundances (proportion of ***N*** in co-cultures with IMP compared to those without) was highest when the initial ratio of ***N*** was low (ratio 100:1) (Fig. 3A). It gradually decreased when the initial ratio of ***N*** increased to 1:1 and 1:100 (slopes > 0, *p*_adj_ = 0.002, linear regression, Table S2). In contrast, this relationship was reversed for ***P***_***conj***_ co-cultures due to the conversion of ***N*** by conjugation (Fig. 3A). The relative difference of the ***N*** population (***N***[***P***_***conj***_]) was significantly higher when fewer ***P***_***conj***_ were initially present (slopes < 0, *p*_adj_ = 0.008, linear regression, Table S2), because the relative number of ***T*** gradually increased as the initial proportion of ***P***_***conj***_ decreased (Fig. S9, *n* = 6).

Another interconnection was found when comparing conjugation efficiencies (i.e., conversion efficiency) and transconjugant abundances in co-cultures exposed to IMP, TET and without antibiotics. The conjugation efficiency (Fig. 3B, *n* = 6) was defined as the ratio of transconjugants divided by all surviving ***N*** (all possible recipients). When the initial producer to non-producer ratio was 1:100, the conjugation efficiency in AB-free co-cultures was lower than co-cultures with IMP, which in turn was lower than those with TET. TET co-cultures had a conjugation efficiency of 1 because all surviving ***N*** were transconjugants. At the 1:1 ratio, the conjugation efficiency in AB-free co-cultures was higher than at the 1:100 ratio and slightly lower compared to co-cultures with IMP, which were lower than TET. When the ratio was 100:1, all ***N*** from the IMP and AB-free co-cultures were transconjugants (***T***, conjugation efficiency of 1), and no ***N*** (***N***_***rem***_ nor ***T***) were observed when with TET. Overall, the conjugation efficiency in IMP and AB-free co-cultures increased with the initial proportion of producers. In contrast, when comparing transconjugant abundances (i.e., convert abundances) (Fig. 3C, *n* = 6) the opposite trend was observed. Despite low conjugation efficiencies in AB-free co-cultures, the abundance of ***T*** was higher compared to IMP and TET regardless of initial ratios (*p*_adj_ < 0.001, two-sided t-test, Table S2). The lowest transconjugant abundances were found among co-cultures exposed to TET and overall ***T*** was more abundant when the abundance of ***N*** was higher. Collectively, the presence of antibiotics decreased transconjugant abundance, especially under TET treatment (Fig. 3C).

### Social conversion by conjugation and chromosomal association are both successful strategies for the impediment of non-cooperators

**Fig. 4:**
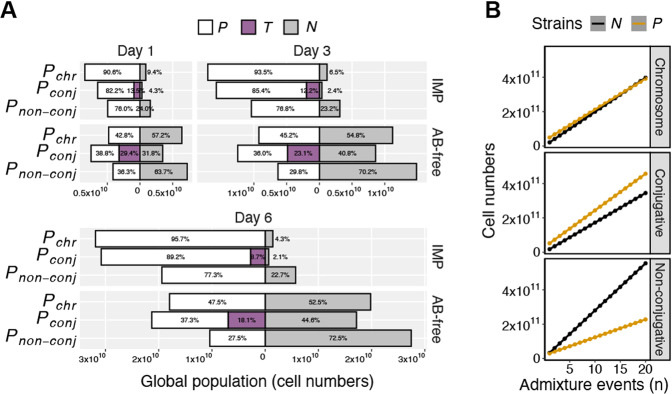
Both social conversion by conjugation and chromosomal association ameliorate non-cooperators. **A** Global populations exposed to imipenem (IMP) and without IMP (AB-free) on day one, from day one to three, and day one to six. ***P*** are KPC-2 producers, either ***P***_***chr***_, ***P***_***conj***_, or ***P***_***non-conj***_ as specified. ***T*** are transconjugants. ***N*** are non-producers. **B** Admixture model where day six global populations not exposed to antibiotics were added with *n* (1 to 20) global populations exposed to IMP. The yellow line is the total number of producers in the global population mix and the black line is the non-producers.

The efficiencies at which non-cooperators were impeded by each of the three different types of cooperative antibiotic resistance producers was evaluated. As shown above (Fig. 3A), the ratio between public goods producers and non-cooperators strongly affects the fitness of non-cooperators. Therefore, in line with previous studies [43, 44], to constitute this effect the co-cultures initiated at the three different ratios (1:100, 1:1, and 100:1) were considered as “subpopulations” within a “global population”. The average of total cell numbers over time were attained for the subpopulations and by summing up the averages of the subpopulations over time, both for those with and without IMP, the cell numbers of global populations were calculated (Fig. S10 and Table S3, *n* = 6). When treated with IMP after one day, fewer cells were found in the global populations with ***P***_***non-conj***_ than those of ***P***_***chr***_ (*p*_adj_ < 0.001). This was a consequence of higher levels of public goods utilization by non-producers and the burden of plasmid carriage in ***P***_***non-conj***_ global populations (Fig. 4A and Table S4). Similar trends were observed until day three and six. The total cell numbers in the global populations with ***P***_***conj***_ after one day of IMP treatment, were lower than those of ***P***_***chr***_ (*p*_adj_ < 0.001), and more similar to ***P***_***non-conj***_, but higher (*p*_adj_ = 0.795). The difference in total cell numbers between global populations with ***P***_***conj***_ and ***P***_***non-conj***_ became more pronounced from day one to day three and more so until day six, and those with ***P***_***conj***_ gradually became comparable to those with ***P***_***chr***_. Yet, in global populations exposed to IMP, while fewer producers (including ***T***) were observed in ***P***_***conj***_ compared to ***P***_***chr***_, fewer non-cooperators were also present in ***P***_***conj***_. In comparison, public goods utilization by non-producers was evidently more pronounced in the global populations with ***P***_***non-conj***_ exposed to IMP than in those with ***P***_***conj***_ and ***P***_***chr***_, where the numbers of producers and non-cooperators were comparable.

As expected, when no antibiotics were present the numbers of both total cells and non-producers were higher overall in all global populations. On day one in the global populations with ***P***_***chr***_ not exposed to antibiotics, the ratio between non-producers and producers was close to 1:1 but slightly more non-producers were present. This seemed to be a stable trend also seen from day one until day three and six. In global populations with ***P***_***non-conj***_, non-producers outnumbered producers. Additionally, in contrast to global populations with ***P***_***chr***_ or ***P***_***non-conj***_, more producers (including ***T***) were observed than non-producers in global populations with ***P***_***conj***_ (day one, *p*_adj_ < 0.001). The total number of producers in ***P***_***conj***_ were also higher compared to ***P***_***chr***_ (day one, *p*_adj_ < 0.001)_*.*_

Overall, producers outcompeted non-producers globally when in the presence of IMP. However, when IMP wasn’t present non-producers were more abundant but at varying levels. The level at which bacteria are exposed to antibiotics in hosts or other environments often fluctuates over time [45]. Therefore, we evaluated how many times an IMP treated global population would have to mix, with global populations that had not been exposed to antibiotics, before the total number of non-producers would surpass the number of producers. This was done by adding the total average cell numbers from the IMP treated global populations with averages from *n* (1 to 20) AB-free global populations (Fig. 4B). We found that any mixing of an IMP treated global population with ***P***_***non-conj***_, with those without IMP, will result in producers being outcompeted regardless of how many. This contrasts with results for the global populations with ***P***_***chr***_ and ***P***_***conj***_. For ***P***_***chr***_ the non-producers became the majority when one IMP exposed global population was mixed with seventeen global populations not exposed to IMP. This didn’t seem to be the case for global populations with ***P***_***conj***_, as the intermixing events kept increasing the proportion of producers due to conjugation. While simplistic, this illustrates that both chromosomal and conjugative plasmid association of *bla*_KPC-2_ can impede non-cooperators globally (Fig. 4B).

### Non-cooperative plasmids can intrude but are unlikely to persist in populations if periodically exposed to β-lactam antibiotics

**Fig. 5:**
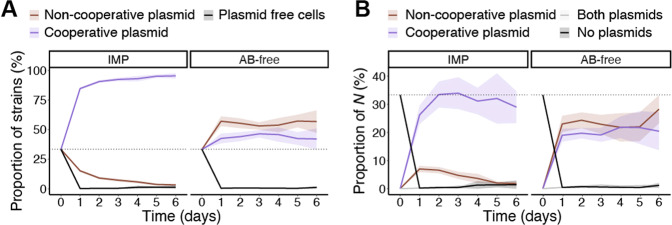
Restricted impact of intrusion by non-cooperative plasmids. **A** Proportions of cells with the cooperative plasmid (***P***_***conj***_), the non-cooperative plasmid (***N***_***conj n-coop***_), and plasmid-free non-producers (***N***) in co-cultures initiated at a 1:1:1 ratio. Colonies were cultivated with imipenem (IMP) or without antibiotics (AB-free) for six days. **B** Proportion of subpopulations of rescued cells of the initial non-producer population either with the cooperative plasmid, the non-cooperative plasmid, both plasmids, or without plasmids. For (**A**) and (**B**), lines are the mean and shaded areas are 95% confidence intervals of the mean (*n* = 10). The dashed lines are the initial proportion of each strain (33.3%).

The above data, based on co-cultures with ***P***_***chr***_, ***P***_***conj***_, or ***P***_***non-conj***_ and ***N*** (Fig.1E, AB-free), suggested that expressing the KPC-2 β-lactamase on its own did not impose much of a fitness burden but carrying a conjugative plasmid did. Yet, conversion by conjugation helped impede non-cooperators, especially when producers were few (Figs. 3 and 4, 1:100). This could imply that a plasmid without the β-lactamase gene (non-cooperative plasmid) could potentially outcompete a producer plasmid (cooperative plasmid). To assess the invasion potential of a non-cooperative plasmid, a non-producer strain with a non-cooperative plasmid (***N***_***conj n-coop***_) was designed. The non-cooperative plasmid was similar to that of ***P***_***conj***_ but did not encode KPC-2. ***N***_***conj n-coop***_ was co-cultured with ***N*** and ***P***_***conj***_ at an initial ratio of 1:1:1 (Fig. 5, *n* = 10). After one day of co-cultivation, without antibiotics, the vast majority of cells (99.6%) carried one of the two plasmids. Just under half carried the cooperative plasmid (43.9%) and just over half the non-cooperative plasmid (55.5%) (Fig. 5A, AB-free). This slight difference was observed among the transconjugants during the first two days. Hereafter, the proportion of the transconjugants that carried the two plasmids became similar (Fig. 5B). Very few cells carried both the non-cooperative and cooperative plasmid, likely due to interference by incompatibility or surface exclusion (0.18% in IMP and 0.43% in AB-free on day one). Although a minor fraction, some ***N*** (0.42% on day one) did not acquire any of the plasmids and their numbers increased gradually during the six days (1.15% on day six). Correspondingly, in the presence of IMP, most cells ended up carrying plasmids (Fig. 5A), of which the vast majority was the cooperative plasmid (84.64% on day one). The non-cooperative plasmid was present at relatively higher proportions on day one (15.16%) and then dropped continuously until the end of the experiment at day six (slope = −2.208, *p*_adj_ < 0.001, linear regression). This trend was different from what was observed in co-cultures without a non-cooperative plasmid where the proportion of rescued ***N***_***ori***_ remained relatively constant after the first couple of days (Fig. 1E, IMP). This implies that the fitness of transconjugants with the non-cooperative plasmid was lower than non-producers without a plasmid (20–40% in Fig. 1E; IMP; 0–20% in Fig. 5B, IMP). To not cooperate was thus a less viable strategy for strains with a non-producer plasmid, compared to not cooperating without a plasmid. A statistical comparison of the averaged proportions of the strains, with or without IMP, after one to six days of co-cultivation, is shown in Table S2.

***P***_***conj***_, ***N***_***conj n-coop***_, and ***N*** were also co-cultured at an initial ratio of 2:1:1, so that the producer to non-producer ratio would be 1:1 (Fig. S11, *n* = 6). As expected, the proportion of cells with the cooperative plasmid was higher than those with the non-cooperative plasmid when IMP was present (Fig. S11, IMP). The same trend was observed in co-cultures without antibiotics (Fig. S11, AB-free). In the presence of IMP, the proportion of non-cooperative plasmids kept decreasing during the six days (slope = −2.074, *p*_adj_ < 0.001, linear regression). By direct comparison with the co-culture data initiated at 1:1 ratio of ***P***_***conj***_ and ***N*** (Fig. S12, *n* = 6), the proportion of non-producers (including ***N***_***conj n-coop***_) was consistently lower in ***P***_***conj***_ and ***N*** co-cultures compared to the co-cultures with the non-cooperative plasmid. This shows that the non-cooperative plasmid initially hindered the invasion and spread of the cooperative plasmid (especially on day one and two), but this effect was no longer detected after day three.

## Discussion

Here, we find that KPC-2 β-lactamases are public goods with extracellular activity that can rescue sensitive non-producers when exposed to above-MIC concentrations of the β-lactam imipenem (Fig. 1, Fig. S3 and Figs. 5–7). Due to the higher copy number of plasmids compared to chromosomes, increased expression levels resulted in higher β-lactamase activity and thus improved rescue of non-producers in co-cultures (Fig. 1). Moreover, rescuing non-producers by public good antibiotic resistance advanced the presence of KPC-2 encoding conjugative plasmids in the community, as the plasmids efficiently transferred to the rescued non-producers. This way, compared to private good tetracycline resistance, plasmids encoding cooperative resistance expanded the potential recipient pool by increasing the number of transconjugants (transconjugant abundance) and the probability of plasmid transfer to new genotypes. Conversely, private good resistance ensured the highest conjugation efficiency as only donors and transconjugants survived (Figs. 2, 3, and Fig. S8).

The experiments were conducted on solid agar medium in line with, and to further extend previous work on cooperative antibiotic resistance [2, 23, 46]. In colonies grown on agar medium, susceptible cells have been shown to be more likely to survive during social protection by β-lactamase producers [23]. For other public goods, the spatial structure has been shown to be an important factor that can exclude non-cooperators due to limited dispersion [1, 47, 48]. Rather than having to reduce the overall β-lactam concentration in a liquid medium, enabling the rescue of non-producers, rescue is likely more efficient in colonies, because β-lactams mainly are hydrolyzed locally spatially creating a steep gradient of β-lactams. Moreover, bacterial colonies on agar medium create a structured environment that resembles bacterial biofilms [8].

When public goods were readily available, non-cooperators thrived and social conversion by conjugation became less pronounced (Fig. 3A). This was likely because a large number of public good producers increase the relative fitness of the non-cooperators [49]. However, when fewer public good producers, which were also plasmid donors, were present social conversion by conjugation was relatively more pronounced, while the fitness of non-cooperators was at its lowest. These data highlight an interconnection between the potential of public good exploitation and the efficiency of social conversion by conjugation (Fig. 3A), and the conjugation efficiencies and transconjugant abundances illustrated an important difference in the transfer dynamics of plasmids when encoding private versus public good antibiotic resistances (Fig. 3B). In the case of private antibiotic resistance, ***N*** were competitive if producers were not the majority. However, because only a few of the potential recipients received the plasmid, the actual number of conjugation events was always low for the private antibiotic resistance. This disclosed that cooperative and private antibiotic resistance, respectively, advanced transconjugant abundance and conjugation efficiency.

We noted that conjugation led to social conversion during antibiotic selection especially when donors/producers initially were rare. This is consistent with previous findings that HGT can enhance inclusive fitness benefits by increasing the relatedness of neighboring cells in a structured environment when public goods production is low [30]. Also, conjugative transfer has been reported to contribute to the initial invasion of cooperative genes [50, 51]. Here we found that non-cooperative plasmids, that did not encode KPC-2, were only slightly more competitive than cooperative plasmids when no antibiotics were present. However, when imipenem was present the non-cooperative plasmids were efficiently outcompeted and the non-producers that persisted over time were those without the non-cooperative plasmid. We observed that competition among non-cooperators that were protected by cooperators disfavors non-cooperative plasmids, thus, they could intrude but were unlikely to persist.

The positive relationship between the number of *bla*_KPC-2_ copies and the rescue of non-producers shown here, implies that the association of public goods specifically elevates the fitness of the replicon it is encoded on: (i) When encoded in a conjugative plasmid, *bla*_KPC-2_ advanced its presence in the community by also rescuing a relatively large number of potential recipients, which subsequently resulted in them acquiring the plasmid. (ii) In contrast, when encoded in the chromosome the metabolic burden of KPC-2 was minimal, and relatively fewer non-producers were rescued. This meant that chromosomally encoded KPC-2 was more privatized and the competitiveness of the associated replicon, the chromosome, was thus also improved. Nonetheless, the expression of chromosomal *bla*_KPC-2_ did lead to some rescue of non-producers and thus supported the survival of competing non-cooperative cells. In contrast, the ***N*** populations in co-cultures with ***P***_***conj***_ were kept at bay due to the fitness cost imposed on them when transconjugants acquired the plasmid via conjugation (Fig. 1E). So, while the burden of carrying plasmids may weaken the competitiveness of the transconjugant producers (Fig. S10), the conjugative conversion will enhance the competitiveness of the original producer population as a similar burden is transferred to transconjugants (Fig. 3). The results thus suggest that public goods antibiotic resistance especially promotes the fitness of replicons that can transfer horizontally. Correspondingly, most β-lactamase genes, including *bla*_KPC-2_, predominantly are found encoded on plasmids [24, 25, 52–56].

At lower concentrations of imipenem, the rescue of non-producers increased (Fig. S13, *n* = 4), and at below-MIC concentrations, a threshold should exist where the fitness of non-producers becomes equal to and subsequently higher than the producers. At this threshold or at lower concentrations, producers may be outcompeted and the cooperative phenotype lost [57]. Nonetheless, our data suggest that social conversion by conjugation will reduce the risk that this occurs, and that utilization of public goods by non-cooperators is less pronounced when *bla*_KPC-2_ is expressed from chromosomes compared to non-conjugative plasmids (Fig. 4).

It has been stated that the existence of conjugative plasmids is paradoxical [58]. This is because (i) conjugation rates often are too low to solely maintain plasmids in a population, and (ii) accessory genes, such as those coding for antibiotic resistance, are less energetically costly when encoded on the chromosome versus conjugative plasmids, as experimentally verified in this study. Various factors and mechanisms are known to help maintain plasmids in their host population including toxin-antitoxins [59] and partitioning systems [60]. Furthermore, co-evolution between plasmid and chromosome can reduce the energetic burden the plasmid inflicts [61], for example, the amelioration of plasmid fitness costs due to compensatory mutations [62]. Intriguingly, as shown here, an advantage of carrying a conjugative plasmid is that the plasmid and the cost it imposes can be transferred to other bacteria, hereby enabling the donor to compete more efficiently in the absence of plasmid-specific selection. This suggests that hosts that have co-adapted with a plasmid may be more competitive than transconjugants of strains where co-adaptation has not taken place.

Recently it was discussed and argued that cooperative enforcement likely is an important mechanism facilitating the evolution of egalitarian cooperation [59]. While cooperative conversion by HGT is a distinctive mechanism of safeguarding cooperation, the overall result—that non-cooperators are *forced* to cooperate—is conceptually similar to cooperative enforcement. In line with this, cooperative conversion by HGT may play a role in facilitating the evolution of egalitarian cooperation amongst bacteria.

## Materials and methods

### Bacterial strains and plasmids

The background of all producer and non-producer strains used in this study was *E. coli* MG1655. A full list of strains and plasmids can be found in Table S5, and genome engineering was performed as described in “Supplementary Materials and Methods”. All strains were cultured in standard Luria-Bertani (LB, VWR International, Germany) medium at 30 °C or 37 °C as specified. When needed, appropriate antibiotics (Sigma-Aldrich, USA) were used at the following concentrations: ampicillin (AMP), 100 µg/ml; imipenem (IMP), 4 µg/ml; tetracycline (TET), 15 µg/ml; and kanamycin (KAN), 50 µg/ml.

### β-lactamase activity assay

A colorimetric assay based on the hydrolysis of nitrocefin was used to determine the total intracellular and extracellular β-lactamase activity in LB broth cultures (grown for 24 h) [23]. 500 µl of 24-h-grown LB broth culture was centrifuged at 3000 × *g* for 15 min, and the obtained supernatant was used to measure the activity of extracellular β-lactamases. To measure intracellular activity, the pellet was resuspended in 500 µl PBS containing 1 mg/ml lysozyme and 2 mM EDTA, and then incubated at 37 °C for 1 h. Thereafter, 2 µl of sample was mixed with 198 µl PBS containing 20 µg nitrocefin in a 96-well plate, and placed in a spectrophotometer to detect and record the OD_490_ value every 5 min. All measurements were repeated three times.

### Flow cytometry

Bacterial cells were counted on a BD FACSAria Illu (BD Biosciences, USA) flow cytometer, the related technical settings are outlined in “Supplementary materials and methods”, and the gating strategy was adapted from our previous study [63]. Briefly, a 488 nm excitation laser and the FITC (530/30 nm band-pass filter) detector were used to detect GFP, a 405 nm excitation laser and the DAPI (450/40 nm band-pass filter) detector were used to detect mTagBFP2, and a 561 nm excitation laser and the (PE)-Texas Red (610/20 nm band-pass filter) detector were used to detect mCherry. Data in Fig. 1E were analyzed using FlowJo software (Tree Star Inc., USA).

### Microscopy

Fluorescent microscopy images were obtained with a Leica Stereo M205FA microscope (Germany). A GFP2 filter (excitation filter was 480/40, emission filter was 510LP) was used to excite sfGFP, and a DsRED filter (excitation filter was 546/10, emission filter was 600/40) was used to excite mCherry. 2560 × 1920 pixels of scan area were acquired with a 0.5x objective during laser irradiation. The images were processed using the ImageJ software [64].

### Statistical analysis

A detailed summary of performed statistical analysis is shown in Table S2. Briefly, the mean value of β-lactamase activity, protection efficiency, and cell number were compared with either analysis of variance (ANOVA) followed by post-hoc Tukey’s test, Welch’s *t*-tests, or linear regression followed by R package “emmeans” [65]. p values were adjusted (*p*_adj_) by Tukey’s test or Benjamini-Hochberg method. To compare the global populations depicted in Fig. 4, values from different days were averaged and then summed across ratios, eventually tested with ANOVA followed by Tukey’s test. Correlations between β-lactamase activity and ***N*** proportions in Fig. 1 were determined using Spearman’s rank-order correlation coefficient (function “rcorr” in R package “Hmisc”). The growth curves were analyzed with the R package Growthcurver [66]. Significance was assumed when *p* or *p*_adj_ were 0.05 or below.

## Supplementary information

Supplementary material

Supplementary Table 2

Supplementary Table 4

## Data Availability

Source data are provided with this paper.

## References

[1] Smith P, Schuster M (2019). Public goods and cheating in microbes. Curr Biol.

[2] Medaney F, Dimitriu T, Ellis RJ, Raymond B (2016). Live to cheat another day: bacterial dormancy facilitates the social exploitation of β-lactamases. ISME J.

[3] Buckling A, Harrison F, Vos M, Brockhurst MA, Gardner A, West SA (2007). Siderophore-mediated cooperation and virulence in *Pseudomonas aeruginosa*. FEMS Microbiol Ecol.

[4] Williams P, Winzer K, Chan WC, Cámara M (2007). Look who’s talking: communication and quorum sensing in the bacterial world. Philos Trans R Soc B Biol Sci.

[5] D’Souza G, Shitut S, Preussger D, Yousif G, Waschina S, Kost C (2018). Ecology and evolution of metabolic cross-feeding interactions in bacteria. Nat Prod Rep.

[6] West SA, Griffin AS, Gardner A (2007). Social semantics: altruism, cooperation, mutualism, strong reciprocity and group selection. J Evol Biol.

[7] Nogueira T, Rankin DJ, Touchon M, Taddei F, Brown SP, Rocha EPC (2009). Horizontal gene transfer of the secretome drives the evolution of bacterial cooperation and virulence. Curr Biol.

[8] Madsen JS, Burmølle M, Hansen LH, Sørensen SJ (2012). The interconnection between biofilm formation and horizontal gene transfer. FEMS Immunol Med Microbiol.

[9] Brouwer MSM, Roberts AP, Hussain H, Williams RJ, Allan E, Mullany P (2013). Horizontal gene transfer converts non-toxigenic *Clostridium difficile* strains into toxin producers. Nat Commun.

[10] Hülter N, Ilhan J, Wein T, Kadibalban AS, Hammerschmidt K, Dagan T (2017). An evolutionary perspective on plasmid lifestyle modes. Curr Opin Microbiol.

[11] Smillie C, Garcillan-Barcia MP, Francia MV, Rocha EPC, de la Cruz F (2010). Mobility of plasmids. Microbiol Mol Biol Rev.

[12] Bennett PM (2008). Plasmid encoded antibiotic resistance: acquisition and transfer of antibiotic resistance genes in bacteria. Br J Pharmacol.

[13] Von Wintersdorff CJH, Penders J, Van Niekerk JM, Mills ND, Majumder S, Van Alphen LB (2016). Dissemination of antimicrobial resistance in microbial ecosystems through horizontal gene transfer. Front Microbiol.

[14] Anjum M, Madsen JS, Espinosa-Gongora C, Jana B, Wiese M, Nielsen DS (2018). A culture-independent method for studying transfer of IncI1 plasmids from wild-type *Escherichia coli* in complex microbial communities. J Microbiol Methods.

[15] World Health Organization. Antimicrobial resistance: global report on surveillance. World Health Organization; 2014. https://apps.who.int/iris/handle/10665/112642.

[16] de Kraker MEA, Stewardson AJ, Harbarth S (2016). Will 10 million people die a year due to antimicrobial resistance by 2050?. PLoS Med.

[17] Bonomo RA, Burd EM, Conly J, Limbago BM, Poirel L, Segre JA (2018). Carbapenemase-producing organisms: a global scourge. Clin Infect Dis.

[18] Rupp M, Fey PD (2003). Extended spectrum β-lactamase (ESBL) - producing *Enterobacteriaceae*. Drugs.

[19] Papp-Wallace KM, Endimiani A, Taracila MA, Bonomo RA (2011). Carbapenems: past, present, and future. Antimicrob Agents Chemother.

[20] Crofts TS, Gasparrini AJ, Dantas G (2017). Next-generation approaches to understand and combat the antibiotic resistome. Nat Rev Microbiol.

[21] Carattoli A (2009). Resistance plasmid families in *Enterobacteriaceae*. Antimicrob Agents Chemother.

[22] Perlin MH, Clark DR, McKenzie C, Patel H, Jackson N, Kormanik C (2009). Protection of *Salmonella* by ampicillin-resistant *Escherichia coli* in the presence of otherwise lethal drug concentrations. Proc R Soc B Biol Sci.

[23] Amanatidou E, Matthews AC, Kuhlicke U, Neu TR, McEvoy JP, Raymond B (2019). Biofilms facilitate cheating and social exploitation of β-lactam resistance in *Escherichia coli*. npj Biofilms Microbiomes.

[24] Wei ZQ, Du XX, Yu YS, Shen P, Chen YG, Li LJ (2007). Plasmid-mediated KPC-2 in a *Klebsiella pneumoniae* isolate from China. Antimicrob Agents Chemother.

[25] Smith Moland E, Hanson ND, Herrera VL, Black JA, Lockhart TJ, Hossain A (2003). Plasmid-mediated, carbapenem-hydrolysing β-lactamase, KPC-2, in *Klebsiella pneumoniae* isolates. J Antimicrob Chemother.

[26] Wolter DJ, Kurpiel PM, Woodford N, Palepou MFI, Goering RV, Hanson ND (2009). Phenotypic and enzymatic comparative analysis of the novel KPC variant KPC-5 and its evolutionary variants, KPC-2 and KPC-4. Antimicrob Agents Chemother.

[27] Yigit H, Queenan AM, Rasheed JK, Biddle JW, Domenech-Sanchez A, Alberti S (2003). Carbapenem-resistant strain of *Klebsiella oxytoca* harboring carbapenem-hydrolyzing β-lactamase KPC-2. Antimicrob Agents Chemother.

[28] Monteiro J, Santos AF, Asensi MD, Peirano G, Gales AC (2009). First report of KPC-2-producing *Klebsiella pneumoniae* strains in Brazil. Antimicrob Agents Chemother.

[29] Leavitt A, Navon-Venezia S, Chmelnitsky I, Schwaber MJ, Carmeli Y (2007). Emergence of KPC-2 and KPC-3 in carbapenem-resistant *Klebsiella pneumoniae* strains in an Israeli hospital. Antimicrob Agents Chemother.

[30] Mc Ginty SÉ, Lehmann L, Brown SP, Rankin DJ (2013). The interplay between relatedness and horizontal gene transfer drives the evolution of plasmid-carried public goods. Proc R Soc B Biol Sci.

[31] Dimitriu T, Lotton C, Beńard-Capelle J, Misevic D, Brown SP, Lindner AB (2014). Genetic information transfer promotes cooperation in bacteria. Proc Natl Acad Sci USA.

[32] Mc Ginty SE, Rankin DJ, Brown SP (2011). Horizontal gene transfer and the evolution of bacterial cooperation. Evolution.

[33] Griffin AS, West SA, Buckling A (2004). Cooperation and competition in pathogenic bacteria. Nature.

[34] Figurski DH, Meyer RJ, Helinski DR (1979). Suppression of cole1 replication properties by the Inc P-1 plasmid RK2 in hybrid plasmids constructed in vitro. J Mol Biol.

[35] Bahl MI, Hansen LH, Goesmann A, Sørensen SJ (2007). The multiple antibiotic resistance IncP-1 plasmid pKJK5 isolated from a soil environment is phylogenetically divergent from members of the previously established α, β and δ sub-groups. Plasmid.

[36] Vial L, Hommais F (2020). Plasmid-chromosome cross-talks. Environ Microbiol.

[37] Gama JA, Zilhão R, Dionisio F (2018). Impact of plasmid interactions with the chromosome and other plasmids on the spread of antibiotic resistance. Plasmid..

[38] Millan AS, Escudero JA, Gifford DR, Mazel DI, MacLean RC (2016). Multicopy plasmids potentiate the evolution of antibiotic resistance in bacteria. Nat Ecol Evol.

[39] Sharma A, Wood KB (2021). Spatial segregation and cooperation in radially expanding microbial colonies under antibiotic stress. ISME J.

[40] Lee IPA, Eldakar OT, Gogarten JP, Andam CP (2022). Bacterial cooperation through horizontal gene transfer. Trends Ecol Evol.

[41] Markley JL, Wencewicz TA (2018). Tetracycline-inactivating enzymes. Front Microbiol.

[42] Musovic S, Dechesne A, Sørensen J, Smets BF (2010). Novel assay to assess permissiveness of a soil microbial community toward receipt of mobile genetic elements. Appl Environ Microbiol.

[43] Chuang JS, Rivoire O, Leibler S (2009). Simpson’s Paradox in a synthetic microbial system. Science.

[44] Ross-Gillespie A, Gardner A, West SA, Griffin AS (2007). Frequency dependence and cooperation: theory and a test with bacteria. Am Nat.

[45] Andersson DI, Hughes D (2014). Microbiological effects of sublethal levels of antibiotics. Nat Rev Microbiol.

[46] Frost I, Smith WPJ, Mitri S, Millan AS, Davit Y, Osborne JM (2018). Cooperation, competition and antibiotic resistance in bacterial colonies. ISME J.

[47] Monaco H, Liu KS, Sereno T, Deforet M, Taylor BP, Chen Y (2022). Spatial-temporal dynamics of a microbial cooperative behavior resistant to cheating. Nat Commun.

[48] Stump SMC, Johnson EC, Klausmeier CA (2018). Local interactions and self-organized spatial patterns stabilize microbial cross-feeding against cheaters. J R Soc Interface.

[49] Asfahl KL, Schuster M (2017). Social interactions in bacterial cell-cell signaling. FEMS Microbiol Rev.

[50] Dewar AE, Thomas JL, Scott TW, Wild G, Griffin AS, West SA (2021). Plasmids do not consistently stabilize cooperation across bacteria but may promote broad pathogen host-range. Nat Ecol Evol.

[51] Bakkeren E, Gül E, Huisman JS, Steiger Y, Rocker A, Hardt WD (2022). Impact of horizontal gene transfer on emergence and stability of cooperative virulence in *Salmonella Typhimurium*. Nat Commun.

[52] Che Y, Yang Y, Xu X, Břinda K, Polz MF, Hanage WP (2021). Conjugative plasmids interact with insertion sequences to shape the horizontal transfer of antimicrobial resistance genes. Proc Natl Acad Sci USA.

[53] Hossain A, Ferraro MJ, Pino R, Dew RB, Moland ES, Lockhart TJ (2004). Plasmid-mediated carbapenem-hydrolyzing enzyme KPC-2 in an *Enterobacter* sp. Antimicrob Agents Chemother.

[54] Miriagou V, Tzouvelekis LS, Rossiter S, Tzelepi E, Angulo FJ, Whichard JM (2003). Imipenem resistance in a *Salmonella* clinical strain due to plasmid-mediated class A carbapenemase KPC-2. Antimicrob Agents Chemother.

[55] Navon-Venezia S, Chmelnitsky I, Leavitt A, Schwaber MJ, Schwartz D, Carmeli Y (2006). Plasmid-mediated imipenem-hydrolyzing enzyme KPC-2 among multiple carbapenem-resistant *Escherichia coli* clones in Israel. Antimicrob Agents Chemother.

[56] Villegas MV, Lolans K, Correa A, Suarez CJ, Lopez JA, Vallejo M (2006). First detection of the plasmid-mediated class a carbapenemase KPC-2 in clinical isolates of *Klebsiella pneumoniae* from South America. Antimicrob Agents Chemother.

[57] Dionisio F, Gordo I (2006). The tragedy of the commons, the public goods dilemma, and the meaning of rivalry and excludability in evolutionary biology. Evol Ecol Res.

[58] Harrison E, Brockhurst MA (2012). Plasmid-mediated horizontal gene transfer is a coevolutionary process. Trends Microbiol.

[59] Gerdes K, Rasmussen PB, Molin S (1986). Unique type of plasmid maintenance function: *Postsegregational* killing of plasmid-free cells. Proc Natl Acad Sci USA.

[60] Nordström K, Molin S, Aagaard-Hansen H (1980). Partitioning of plasmid R1 in *Escherichia coli*, I. Kinetics of loss of plasmid derivatives deleted of the *par* region. Plasmid..

[61] Dahlberg C, Chao L (2003). Amelioration of the cost of conjugative plasmid carriage in *Eschericha coli* K12. Genetics.

[62] Hall JPJ, Wright RCT, Harrison E, Muddiman KJ, Wood AJ, Paterson S (2021). Plasmid fitness costs are caused by specific genetic conflicts enabling resolution by compensatory mutation. PLoS Biol.

[63] Pinilla-Redondo R, Olesen AK, Russel J, de Vries LE, Christensen LD, Musovic S (2021). Broad dissemination of plasmids across groundwater-fed rapid sand filter microbiomes. MBio..

[64] Schindelin J, Arganda-Carreras I, Frise E, Kaynig V, Longair M, Pietzsch T (2012). Fiji: an open-source platform for biological-image analysis. Nat Methods.

[65] Lenth R, Singmann H, Love J, Buerkner P, Herve M. emmeans: Estimated marginal means, aka least-squares means. R Packag version. 2018;1:3.

[66] Sprouffske K, Wagner A (2016). Growthcurver: an R package for obtaining interpretable metrics from microbial growth curves. BMC Bioinform.

