# Supplementary material for: Cooperative antibiotic resistance facilitates horizontal gene transfer
Source: ISME J. 2023 Mar 22;17(6):846–54. doi: 10.1038/s41396-023-01393-1 (PMC10203111; doi:10.1038/s41396-023-01393-1)
Supplement: Supplementary file 1 — Supplementary material [file 41396_2023_1393_MOESM1_ESM.pdf]

Title: Cooperative antibiotic resistance facilitates horizontal gene transfer

Authors: Qinqin Wang[1], Shaodong Wei[2], Ana Filipa Silva[1], Jonas Stenløkke Madsen[1,]*

[1] Section of Microbiology, Department of Biology, University of Copenhagen, 2100 Copenhagen, Denmark

[2] National Food Institute, Technical University of Demark, Kemitorvet 202, 2800 Kgs Lyngby, Denmark

* Corresponding author: jsmadsen@bio.ku.dk

Supplementary materials and methods

Genome engineering of bacterial strains and plasmids

Molecular engineering was generally performed following protocols by Green and Sambrook (1) and using mini-Tn7-based (2) and lambda-red-based (3) approaches. Primers used for strain construction and verification are listed in **Table S6**. Primers were synthesized by and purchased from TAG Copenhagen A/S (Denmark). The design and construction of $\boldsymbol{P_{chr}}$, pKJK5-*sfGFP*-*bla*$_{KPC-2}$ and pKJK5$_{NC}$-*sfGFP*-*bla*$_{KPC-2}$ are described in a previous study (4). $\boldsymbol{P_{conj}}$ was constructed by complementing the chromosome of *E. coli* MG1655 with P$_{lpp}$-*mTagBFP2* using the vector pGRG36-P$_{lpp}$-*mTagBFP2* following protocols previously described (4). Plasmid pGRG36-P$_{lpp}$-*mTagBFP2* was constructed as follows: Primers *bfp*-F/R were used to amplify the *mTagBFP2* gene from vector mTagBFP2-pBAD (Addgene plasmid #54572) (5), and primers PGRG36KanPlpp-1-F/R and PGRG36KanPlpp-2-F/R were used to amplify plasmid pGRG36-*mCherry* (6), excluding the *mCherry* gene. The three fragments were fused together using the NEBuilder HiFi DNA Assembly Cloning Kit (NEB #E5520, UK) according to the manufacturer's protocol. Next, pKJK5-*sfGFP*-*bla*$_{KPC-2}$ was transformed into *E. coli* MG1655-*mTagBFP2* by electroporation. $\boldsymbol{P_{non\text{-}conj}}$ was constructed by complementing *E. coli* MG1655-*mTagBFP2* with pKJK5$_{NC}$-*sfGFP*-*bla*$_{KPC-2}$. ***N*** was constructed by complementing the chromosome of *E. coli* MG1655 with *mCherry* using the vector pGRG36-*mCherry* (6) and following the mini-Tn7-based protocols (2). Non-cooperative plasmid pKJK5-*mTagBFP2* was constructed by complementing pKJK5-*att*Tn7 with P$_{A1-O4/O3}$-*mTagBFP2* using the vector pGRG36-P$_{A1-O4/O3}$-*mTagBFP2* following protocols previously described (4). Plasmid pGRG36-P$_{A1-O4/O3}$-*mTagBFP2* was constructed as follows: Primers PGRG36-PA1-F/R were used to amplify the P$_{A1-O4/O3}$ promoter from plasmid pKJK5-*sfGFP*-*bla*$_{KPC-2}$, and primers PGRG36PA10403-*bfp*-F/R, PGRG36PA1-*bfp*-1-F/PGRG36KanPlpp-1-R, PGRG36KanPlpp-2-F/PGRG36PA1-2-R and PGRG36PA1-3-F/R were used to amplify fragments *mTagBFP2*

and PGRG36 with corresponding homologous overhangs. Next, all fragments were fused together using the NEBuilder HiFi DNA Assembly Cloning Kit to generate pGRG36-$P_{A1\text{-}O4/O3}$-*mTagBFP2* following the manufacturer's protocol. $\boldsymbol{N_{conj\ n\text{-}coop}}$, and $\boldsymbol{P_{conj}}$ used in non-cooperative plasmid co-cultures were constructed by conjugative transfer of plasmids pKJK5-*mTagBFP2* and pKJK5-*sfGFP*-$bla_{KPC\text{-}2}$ into wild-type *E. coli* MG1655 for fluorescence specificity.

Determining minimal inhibitory concentrations (MIC)

In this study, the MIC of all strains was determined following the guidelines of Hancock *et al.* (7). For the broth dilution method, suspensions with 100 µl of 5 x 10^5 CFU/ml cells were used per control well in a 96-well microtiter plate. For the agar dilution method, suspensions with 10^4 CFU cells were used for each spot. Agar plates and 96-well plates were incubated at 37 °C for 16 hours. The MIC value was measured by spectrophotometry and the lowest antibiotic concentration that inhibited visible bacterial growth was regarded as the MIC. The experiments were repeated three times independently.

Growth curve analysis

E. coli strains $\boldsymbol{P_{chr}}$, $\boldsymbol{P_{conj}}$, $\boldsymbol{P_{non\text{-}conj}}$, and $\boldsymbol{N}$ were grown overnight in LB broth with appropriate antibiotics at 37°C, and then 1:10^3 dilutions were prepared with LB broth. Next, 200 µl of the dilutions were distributed to each well of the 96-well microtiter plate and incubated for 15 hours in a spectrophotometer (Bio Tek ELx808™ Absorbance Microplate Reader,USA) at 37°C. To measure bacterial growth, the microtiter plate was shaken continuously and optical density at 600 nm wavelength was recorded every 15 minutes. LB broth without bacteria was used as negative control. Six biological replicates were performed for each sample.

Co-culture experiments

The co-culture experiments performed in this study are depicted in **Fig. S2**. First, producers (***P_{chr}***, ***P_{conj}***, ***$P_{non\text{-}conj}$***) and non-producer (***N***) were grown in LB broth overnight at 37 °C, then washed twice with PBS, and the optical density OD_{600} of the bacterial solution was then adjusted to 0.2. Next, the producers and non-producer were mixed in three different ratios (1:100, 1:1, 100:1). Then, 10 µl of the mixed bacterial solution was inoculated on the LB agar plates with or without antibiotic (0.6 µg/ml imipenem or 7 µg/ml tetracycline) in triplicates. The co-cultures were incubated at 37°C. Last, to monitor bacterial growth, data were collected daily for six days. On each day, colonies were imaged by the fluorescence microscope and cell numbers were obtained by flow cytometry.

Similarly, in experiments with non-cooperative plasmids, overnight cultures of producer (***P_{conj}***) and non-producers (***$N_{conj\ n\text{-}coop}$*** and ***N***) were washed twice with PBS and the bacterial solution was adjusted to OD_{600} = 0.2. Next, the three were mixed in ratios 1:1:1 and 2:1:1. 10 µl of the mixture was inoculated onto LB agar plates with or without antibiotic (0.6 µg/ml imipenem), in triplicates. Last, the co-cultures were incubated for six days at 37°C, and cell numbers were collected by flow cytometry every day.

Quantification of conjugation events

Unique conjugation events were quantified manually based on fluorescence microscope imaging of colonies. Non-producer linages in the colonies were first distinguished based on *mcherry* expression, followed by counting the presence of several contiguous or clustered radial patches of *sfGFP* expression in the non-producer population. These transconjugant patches (expressing both *mcherry* and *sfGFP*) were depicted as yellow in the colony images. If transconjugant patches were spatially separated, they were considered to represent a hypothetically unique genotype that originated from a single parent cell. The results were used to estimate the number of conjugation events in each colony.

Techniques settings and sample preparation for flow cytometry

Bacterial cell counts were performed on a BD FACSAria Illu (BD Biosciences) flow cytometer with a 70-µm nozzle. The detection thresholds for forward scatter (FSC) and side scatter (SSC) were set to 200. Data was acquired and analyzed using the BD FACSDiva software v.6.1.3.

The entire bacterial colonies that were grown on agar plates were scraped off and mixed into 1 ml PBS. Hereafter the samples were diluted n-fold in PBS until reaching ~3000 evt/s at a flow rate of 1 (~10 µl/min, https://www.bdbiosciences.com/content/dam/bdb/marketing-documents/BD_FACSAria_III_User_Guide.pdf). Each sample was quantified for 1 minute. The number of events collected by relevant gates (X) was multiplied by the dilution factor (n) to obtain the total cell number of corresponding subpopulation in the entire colony (formulated as $\frac{X \times n \times 1\,ml}{10\,\mu l}$). The sum of all monitored producers (including transconjugants) and non-producers (including carrying non-cooperative plasmids) was enumerated as the total cell number within each colony. Blue, green, and red fluorescent bacterial cells were gated on bivariate contour plots using areas of DAPI, FITC, and PE-Texas Red, respectively (**Fig. S4**). The "Green FP" (green), "MCherry" (red), "Blue FP" (blue), and "transconjugants" (purple) gates were used to count producers, non-producers, cooperative plasmid, non-cooperative plasmid, and transconjugants, respectively. In addition to having *sfGFP* on the plasmid, producers $\boldsymbol{P_{conj}}$ and $\boldsymbol{P_{non\text{-}conj}}$ also had *mTagBFP2* on the chromosome, enabling us to distinguish the producers from the transconjugants. The transconjugants were obtained by FITC and PE-Texas Red bivariate gates as the non-producers with red fluorescence received the plasmids with green fluorescence. Non-producers with the non-cooperative plasmid were numerated using the DAPI and FITC bivariate gates since the non-cooperative plasmids encoded *mTagBFP2*.

Supplementary figures and tables

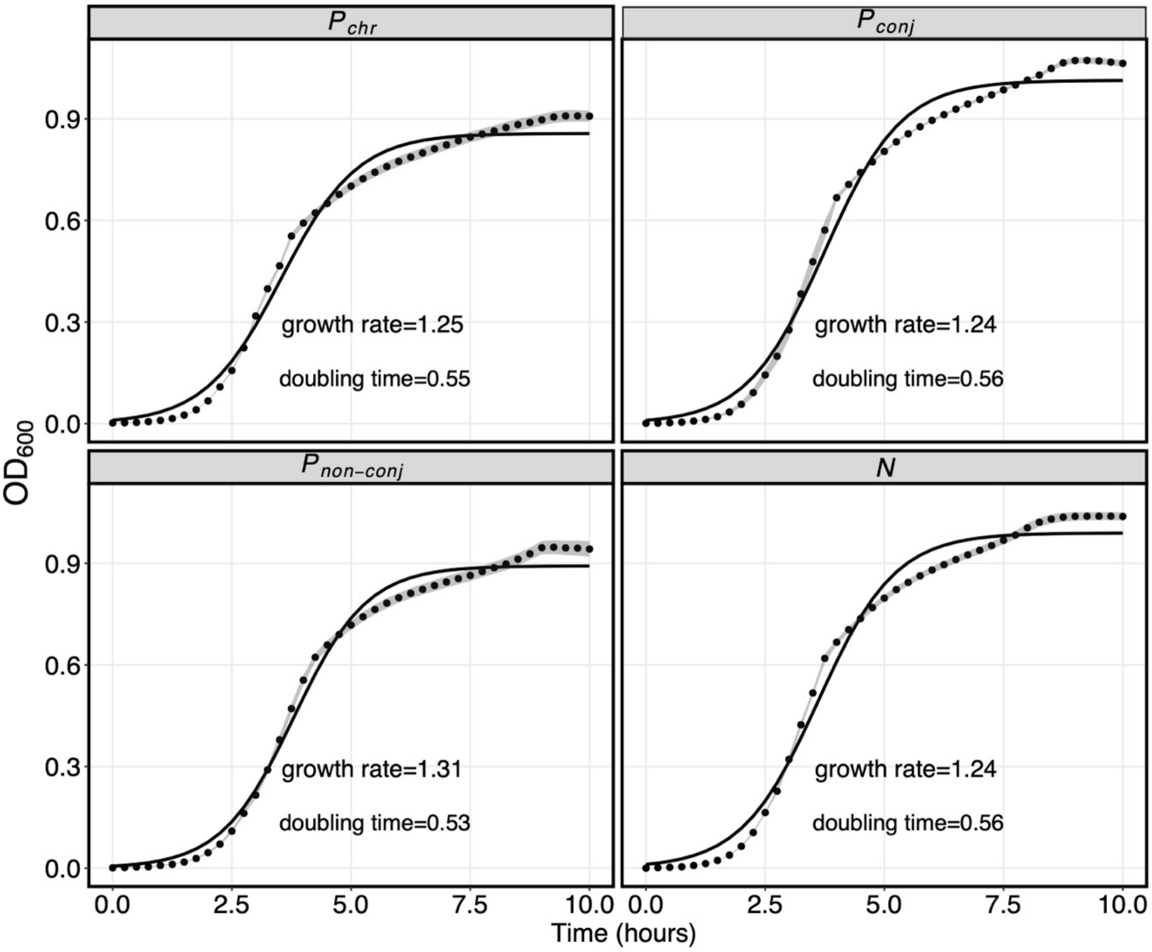

Fig. S1. Growth curve of used strains. Strains (*E. coli* MG1655-*mcherry* [***N***], *E. coli* MG1655-*sfGFP-bla*$_{KPC-2}$ [$\boldsymbol{P_{chr}}$], *E. coli* MG1655-*mTagBFP2*/pKJK5-*sfGFP-bla*$_{KPC-2}$ [$\boldsymbol{P_{conj}}$] and *E. coli* MG1655-*mTagBFP2*/pKJK5$_{NC}$-*sfGFP-bla*$_{KPC-2}$ [$\boldsymbol{P_{non-conj}}$]) were grown for 10 h in LB broth at 37 °C. The X axis is time in hours, and the Y axis is the optical density at 600 nm (OD_{600}). Each colored dot is the averaged OD_{600} values at a time point based on six replicates and the black line is the fitted growth curve with the R-package Growthcurver (8). Shaded area is the standard deviation of the actual OD_{600} at a time point. Four strains showed similar growth rate (n = 6), except that $\boldsymbol{P_{non-conj}}$ was slightly higher in growth rate than others ($p_{adj} < 0.001$, two-sided t-test).

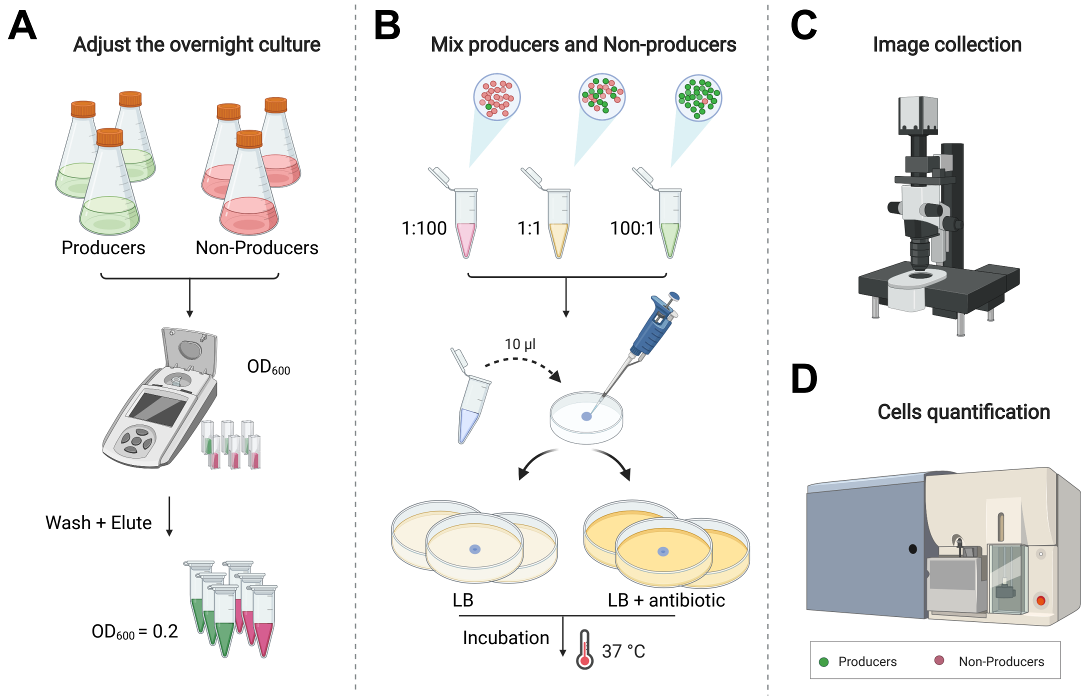

Fig. S2. Diagram of the experimental setup used in the study. (a) Culture preparation. Producers and non-producers were grown overnight, then washed and diluted in PBS to a final cell density of OD_{600} = 0.2. (b) Strain co-cultivation. Producers and non-producers were mixed in three different ratios (1:100, 1:1, 100:1). Then 10 µl of the mixed co-cultures were added on top of LB plates with or without 0.6 µg/ml imipenem. Each condition was performed in triplicate. Co-cultures were incubated at 37°C for six days and data was collected every day. (c-d) Data collection. On each day, colonies were imaged by fluorescence microscopy (Leica Stereo M205FA), and cell numbers were counted by flow cytometry (BD FACSAria IIIu). Figures were created with BioRender.com.

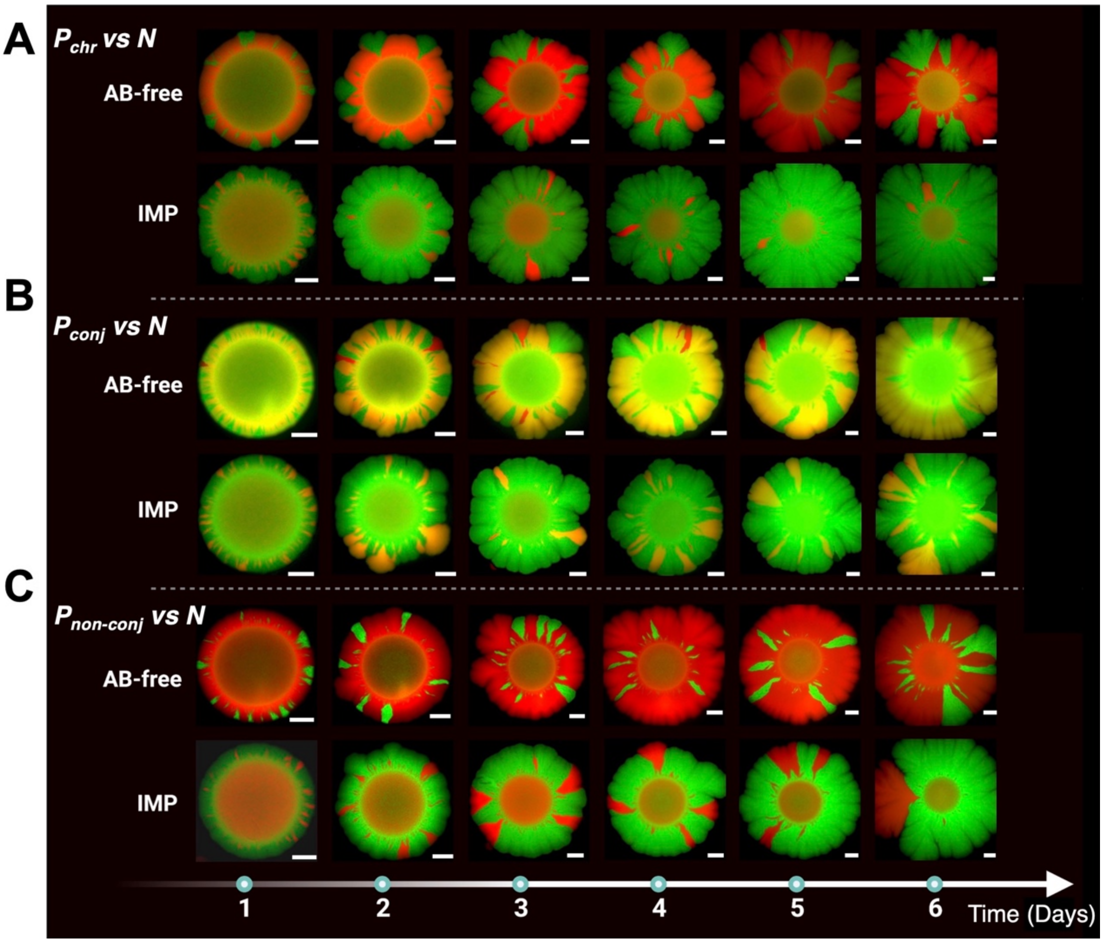

Fig. S3. Spatially extended colonies initiated at a 1:1 ratio of KPC-2 producers to non-producers. Colonies were grown with imipenem (IMP) or without antibiotics (AB-free) for six consecutive days and imaged by fluorescence microscopy. β-lactamases producers (***P_{chr}***, ***P_{conj}***, and ***$P_{non\text{-}conj}$***, green) were mixed with non-producers (***N***, red) at an initial ratio of 1:1. Transconjugants (***T***) are depicted in yellow. White scale bars represent 5 mm.

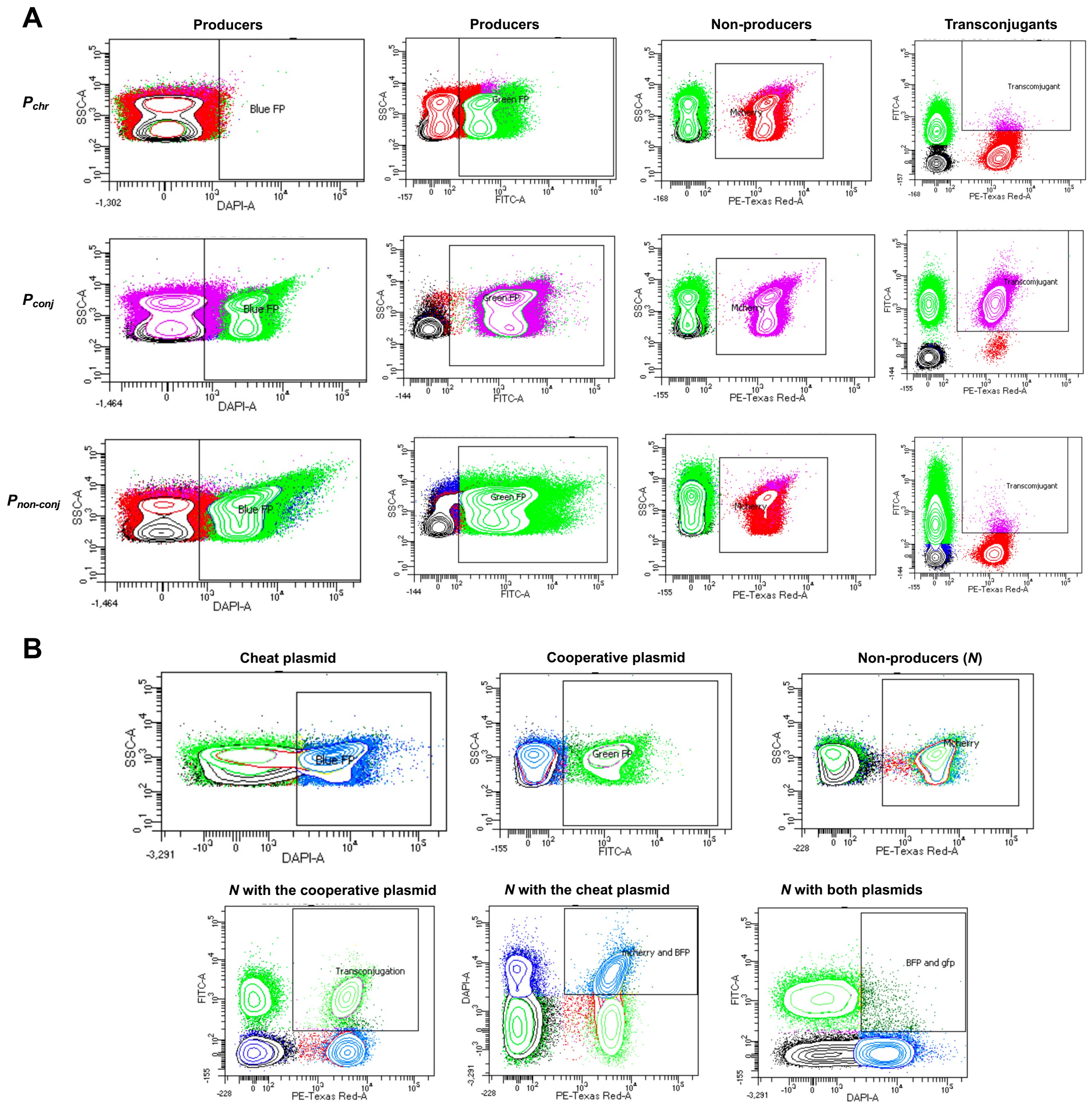

Fig. S4. Gating strategies for flow cytometry. (A) Examples depicted are scatter plots from experiments where β-lactamase producers and non-producers were co-cultivated at an initial ratio of 1:1 after one day without antibiotics. The first and second columns monitored producer cells with DAPI and FITC, respectively. The third column monitored non-producer cells with PE-Texas Red for Red fluorescence (see Methods for specific excitation laser and filter parameters). The fourth column shows transconjugant cells with both red and green fluorescence (FITC and PE-Texas Red). (B) Examples depicted are scatter plots from experiments where cells harboring the cooperative plasmid, non-cooperative plasmid, and without plasmids were co-cultivated at an initial ratio of 1:1:1 after one day without antibiotics. (i) non-cooperative plasmid (DAPI); (ii) cooperative plasmid (FITC); (iii) non-producer (PE-Texas Red); (iv) non-producer with cooperative plasmid (cooperative transconjugant, FITC and PE-Texas Red); (v) non-producer with non-cooperative plasmid (non-cooperative

transconjugant, DAPI and PE-Texas Red); (vi) non-producer with both plasmids (DAPI and FITC).

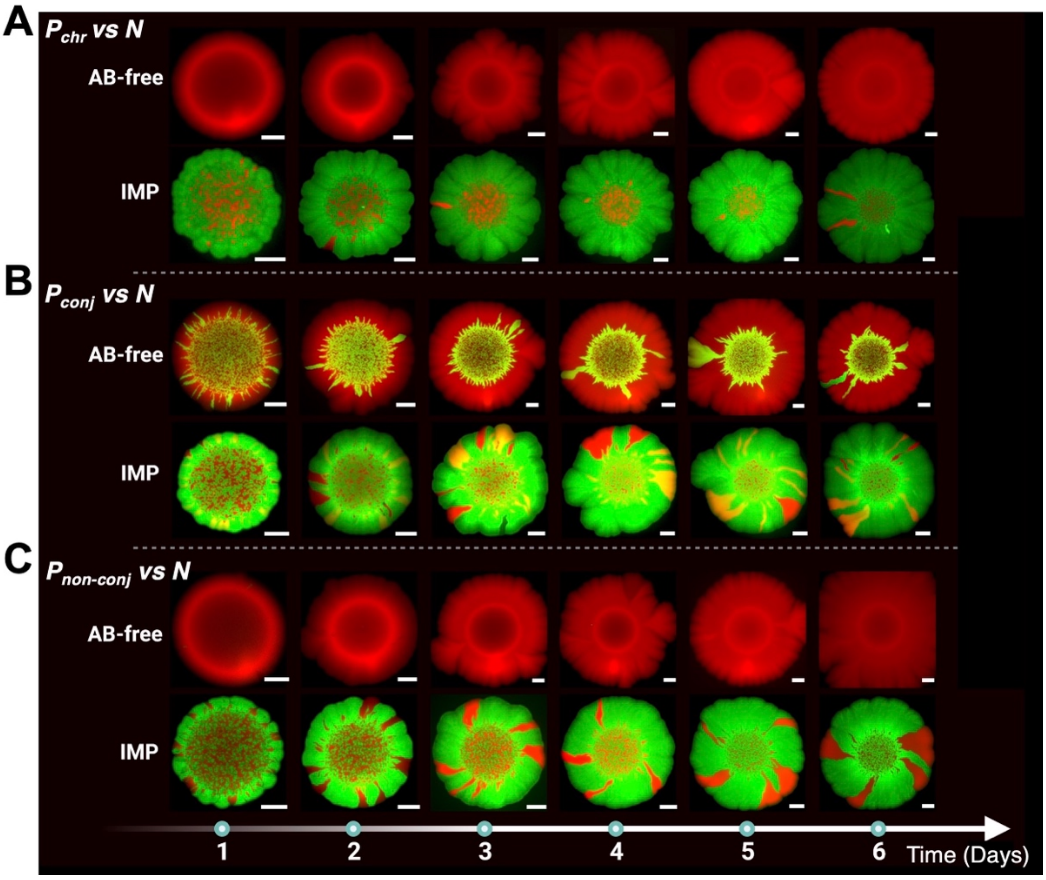

Fig. S5. Spatially extended colonies initiated at a 1:100 ratio of KPC-2 producers to non-producers. Colonies were grown with imipenem (IMP) or without antibiotics (AB-free) for six consecutive days and imaged by fluorescence microscopy. β-lactamases producers (***P***$_{chr}$, ***P***$_{conj}$, and ***P***$_{non\text{-}conj}$, green) were mixed with non-producers (***N***, red) at an initial ratio of 1:100. Transconjugants (***T***) are depicted in yellow. White scale bars represent 5 mm.

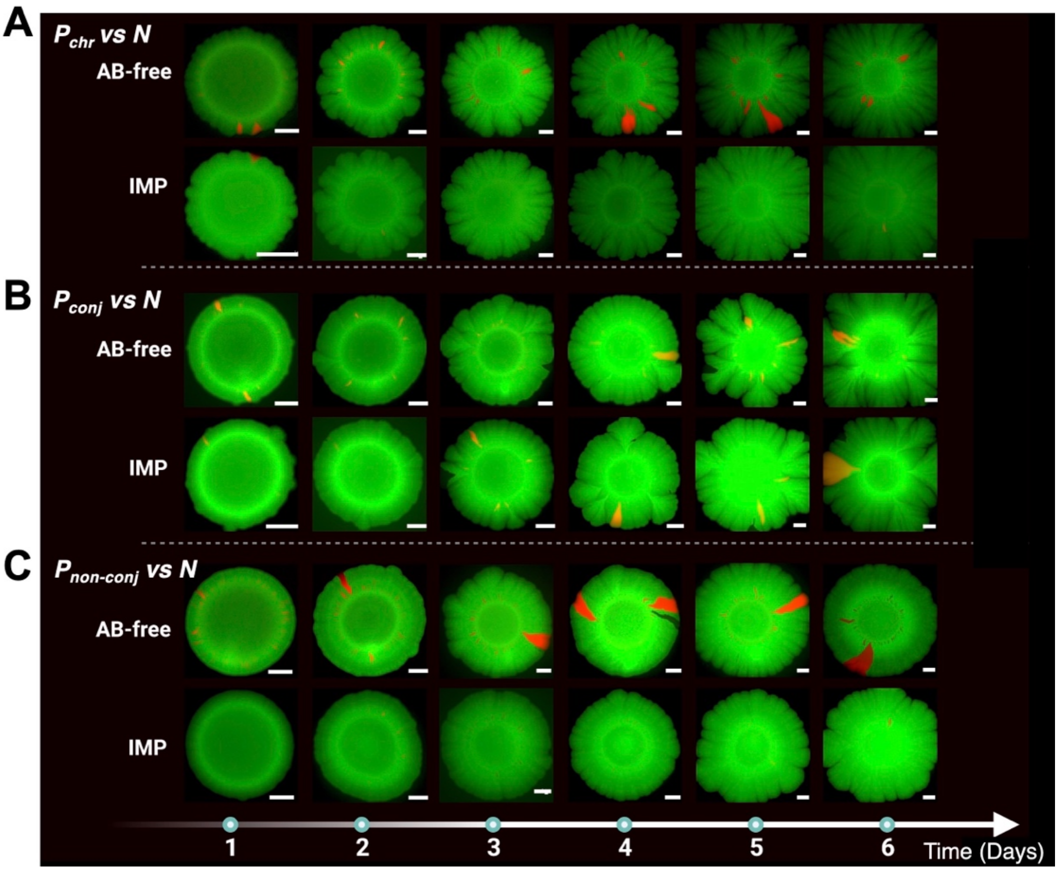

Fig. S6. Spatially extended colonies initiated at a 100:1 of KPC-2 producers to non-producers. Colonies were grown with imipenem (IMP) or without antibiotics (AB-free) for six consecutive days and imaged by fluorescence microscopy. β-lactamases producers (***P**chr*, ***P**conj*, and ***P**non-conj*, green) were mixed with non-producers (***N***, red) at an initial ratio of 100:1. Transconjugants (***T***) are depicted in yellow. White scale bars represent 5 mm.

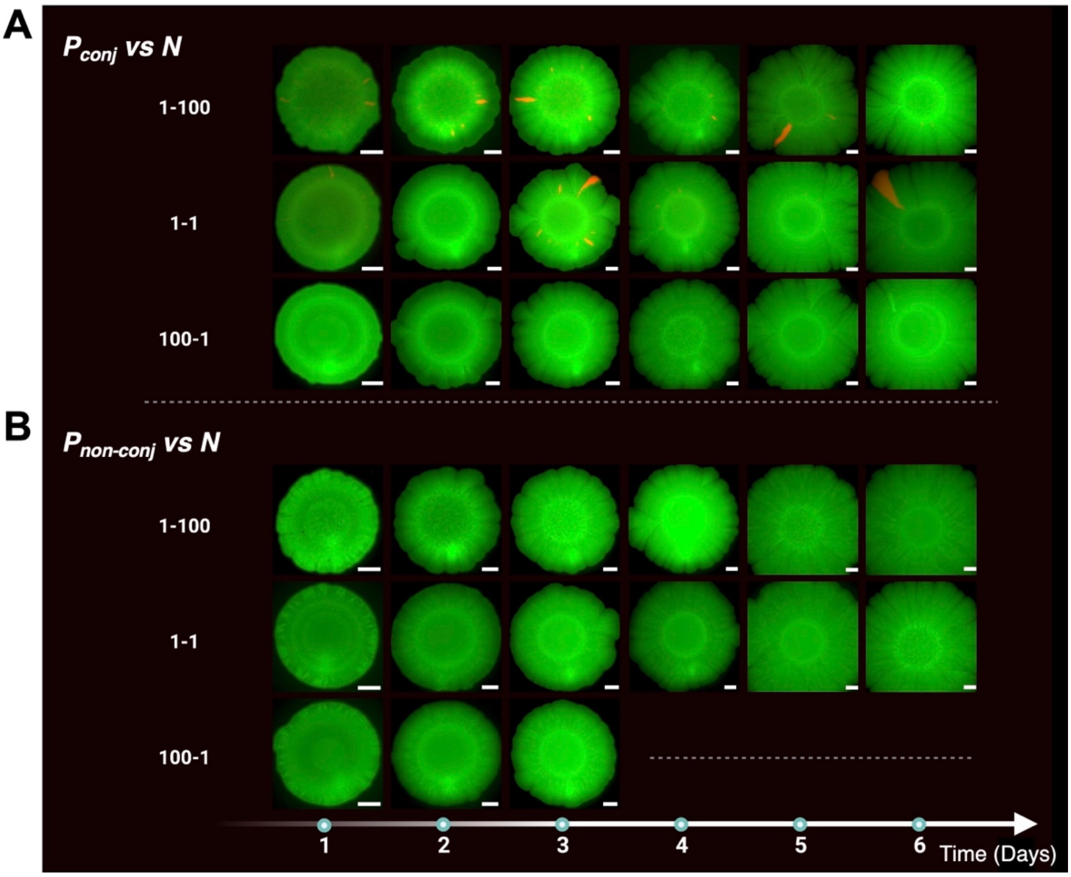

Fig. S7. Spatially extended colonies grown with tetracycline. Colonies were grown with tetracycline for six consecutive days and imaged by fluorescence microscopy. The tetracycline resistant β-lactamase producers ($\boldsymbol{P_{conj}}$, and $\boldsymbol{P_{non\text{-}conj}}$, green) were mixed with tetracycline sensitive non-producers (***N***, red) at initial ratios of 1:100, 1:1, and 100:1. Transconjugants (***T***) are depicted in yellow. The 100:1 ratio co-cultures were followed for only three days. White scale bars represent 5 mm.

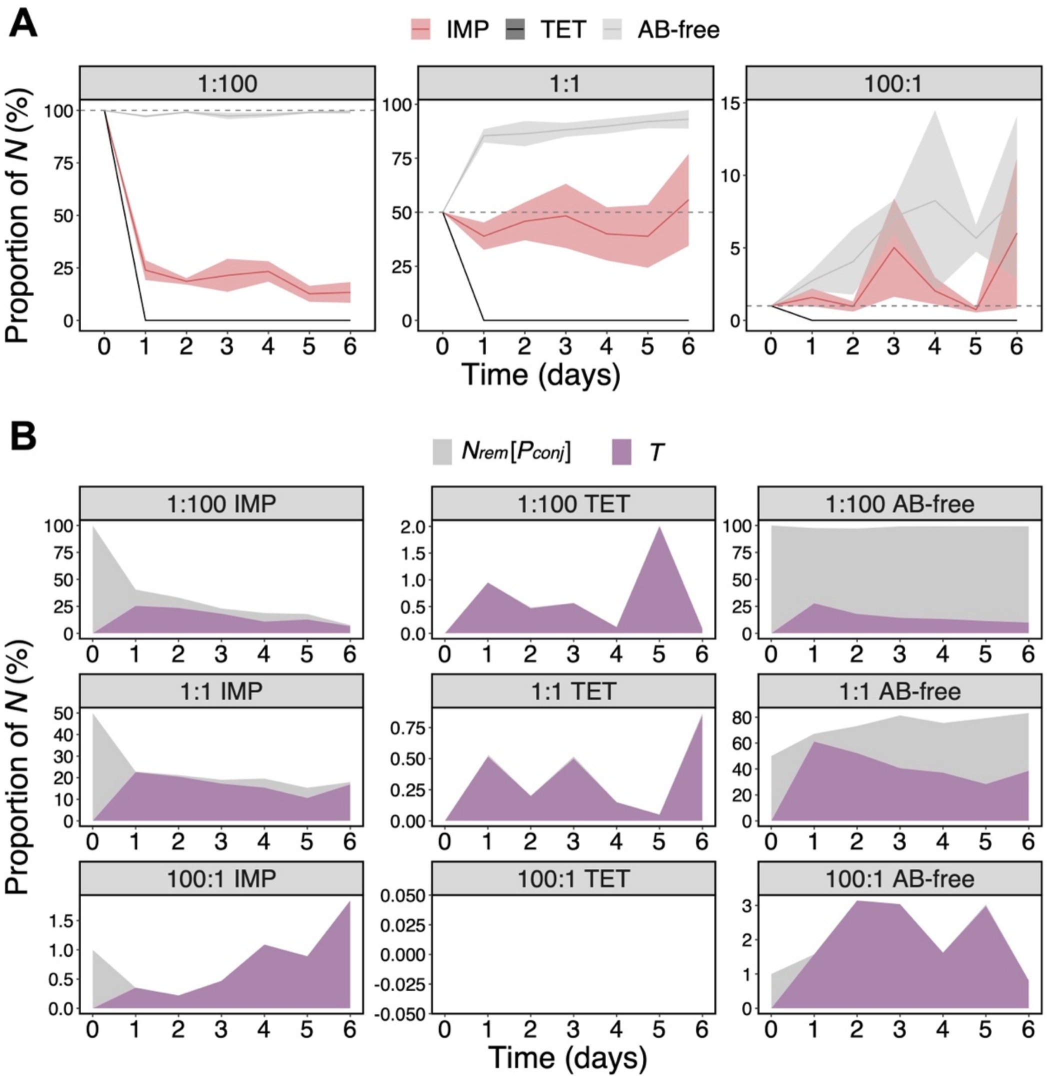

Fig. S8. Proportions of non-producers when co-cultured with cooperative and private antibiotic resistances encoded on non-conjugative and conjugative plasmids. (A) Proportion of non-producers (***N***) in $P_{non\text{-}conj}$ co-cultures with imipenem (IMP, red), tetracycline (TET, black), or without antibiotics (AB-free, gray) grown for six days. Lines and shaded areas show the mean and the 95% confidence interval of the mean (n = 6), respectively. (B) Proportion of remaining non-producers (N_{rem}) and transconjugants (***T***) in P_{conj} co-cultures with imipenem (IMP, red), tetracycline (TET, black), or without antibiotics (AB-free, gray). P_{conj} and ***N*** were mixed at initial ratios of 1:100, 1:1 and 100:1 and grown for six days with imipenem (IMP), tetracycline (TET), or without antibiotics (AB-free). Lines and shaded areas show the mean and the standard error of the mean of replicates (n = 6), respectively. The transconjugants (***T***, orchid) and plasmid-free non-producers (N_{rem}, gray) are shown.

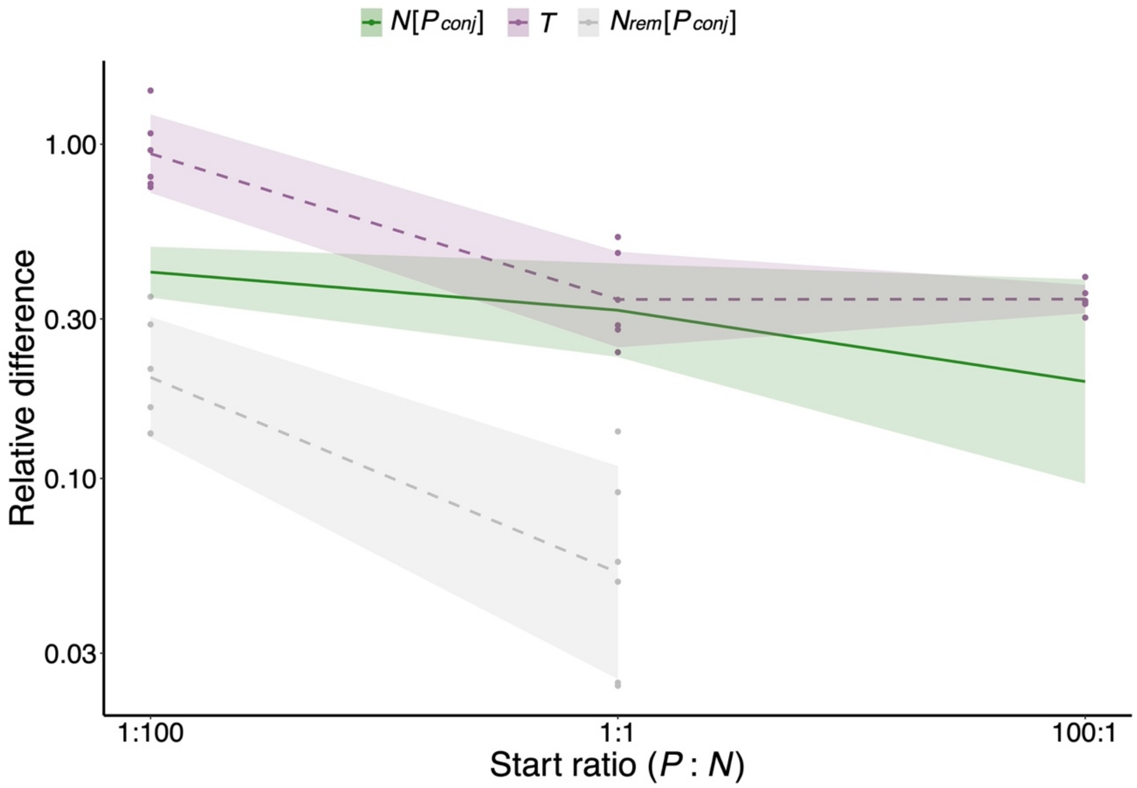

Fig. S9. Relative differences (IMP/AB-free) in abundances of non-producer populations (***N***) when cultivated with [$\boldsymbol{P_{conj}}$] in the presence of imipenem (IMP) and without antibiotics (AB-free), started at different initial producer/non-producer ratios (100:1, 1:1, and 1:100). ***N***[$\boldsymbol{P_{conj}}$] are original non-producer population from $\boldsymbol{P_{conj}}$ co-cultures. ***T*** are transconjugants and $\boldsymbol{N_{rem}}$[$\boldsymbol{P_{conj}}$] are the remaining non-producer population ($\boldsymbol{N_{rem}}$) the ***N*** which have not acquired the plasmid. The lines and shaded areas respectively correspond to the mean of data points (n = 6) and 95% confidence interval of the mean.

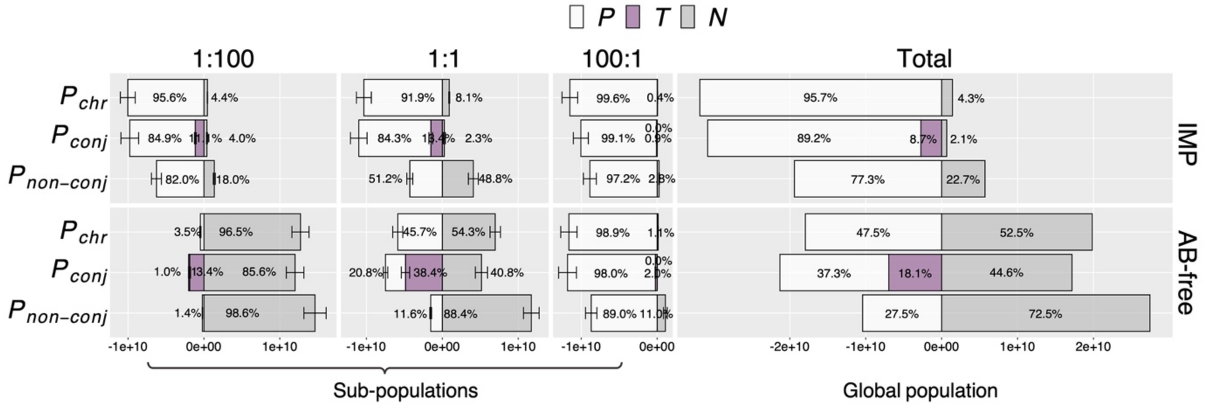

Fig. S10. Total averaged cells numbers in sub-populations and global populations from day one until day six. The total averaged cell numbers (from day one to six) of KPC-2 producers (***P***), transconjugants (***T***) and non-producers without the plasmid (***N***) in co-cultures initiated at ratios of 1:100, 1:1, and 100:1 (sub-populations) of producers (P_{chr}, P_{conj}, or $P_{non\text{-}conj}$) to ***N*** both with imipenem (IMP) and without (AB-free). Error bars are standard error of the mean (n = 6). (Right) Total number of cells in global populations, which are the accumulated averaged numbers of cells from the three individual populations (1:100, 1:1, and 100:1) with IMP and AB-free.

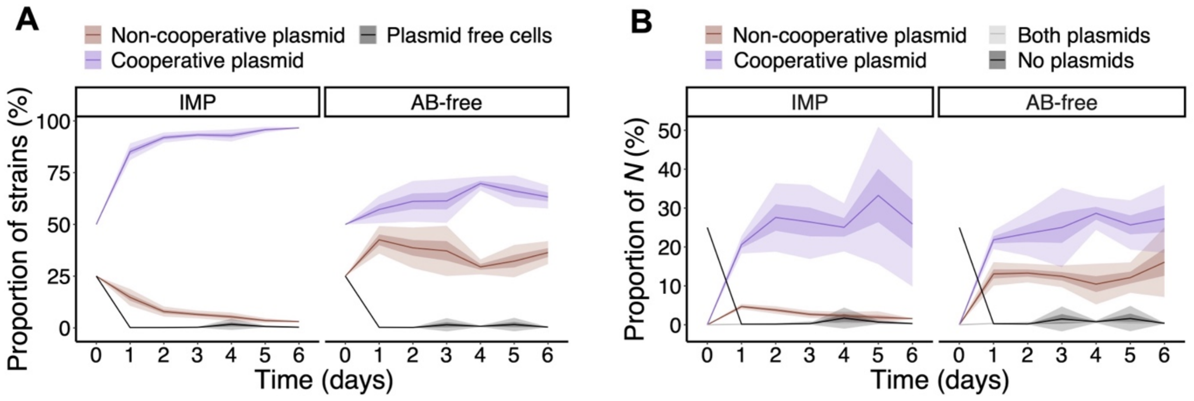

Fig. S11. Restricted impact of intrusion by non-cooperative plasmids in co-cultures initiated at a 2:1:1 ratio of P_{conj}, $N_{conj\ n\text{-}coop}$, and N. (A) Proportions of cells with the cooperative plasmid, the non-cooperative plasmid, and plasmid free cells in co-cultures initiated at a 2:1:1 ratio of P_{conj}, $N_{conj\ n\text{-}coop}$, and N. Colonies were cultivated with imipenem (IMP) or without antibiotics (AB-free) for six days. (B) Proportion of subpopulations of rescued cells of the initial non-producer population either with the cooperative plasmid, the non-cooperative plasmid, both plasmids or without plasmids. For (A) and (B), lines are the mean and shaded areas are 95% confidence interval of the mean (n = 6).

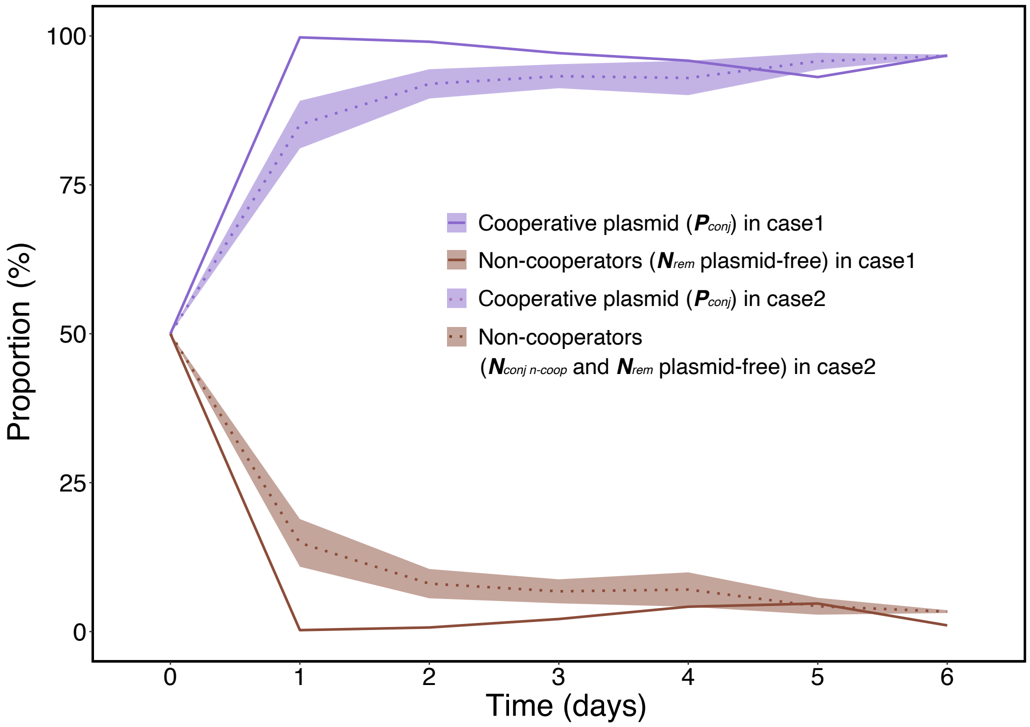

Fig. S12. The proportion of non-producers was consistently lower in P_{conj} and N co-cultures compared to co-cultures with the non-cooperative plasmid. The proportion of transconjugants with either the cooperative or the non-cooperative plasmid during six days of co-cultivation. Two cases of co-cultivation with P_{conj} under IMP treatment were performed, where case 1 is P_{conj} and N were mixed in the ratio of 1:1, and case 2 is P_{conj}, $N_{conj\ n\text{-}coop}$ and N were mixed in the ratio of 2:1:1. The horizontal axis is the number of days. The lines and shaded areas respectively correspond to the mean of data points (n = 6) and 95% confidence interval of the mean.

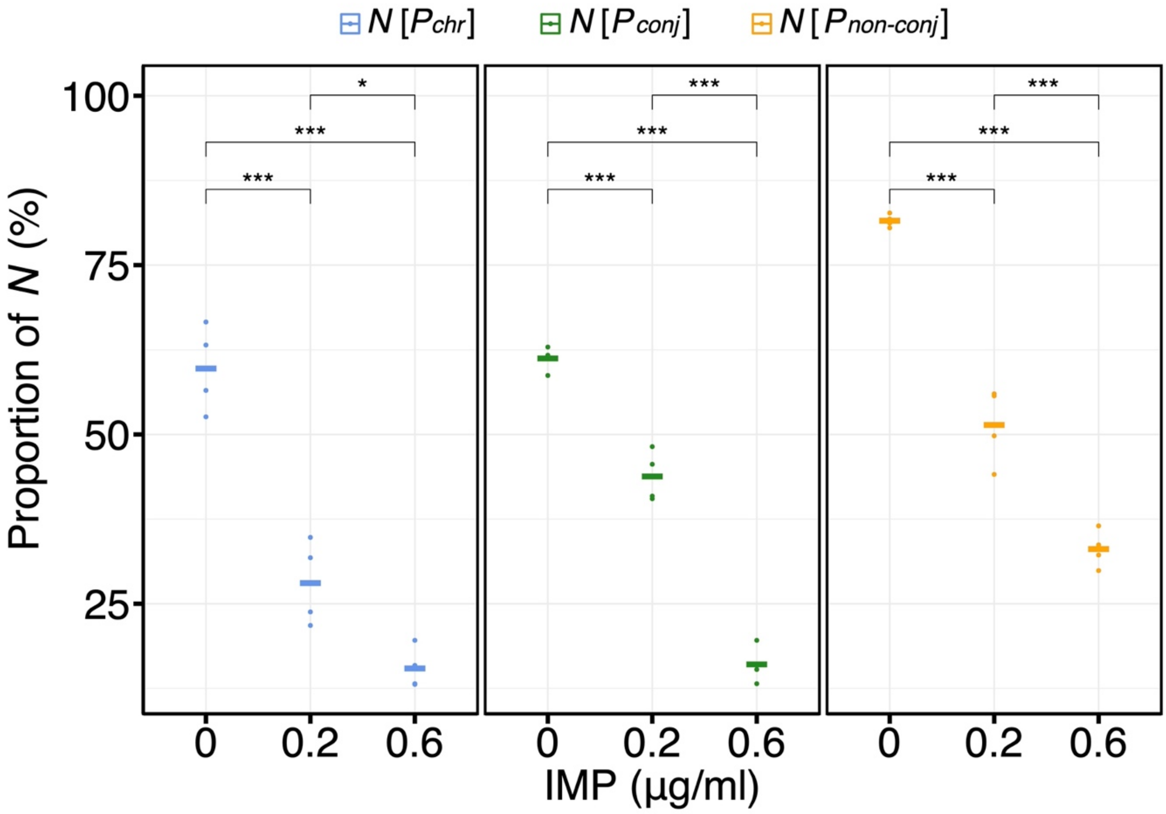

Fig. S13. Higher non-producer rescue at lower imipenem concentrations. The proportion of original non-producers (including transconjugants) after one day of co-cultivation with $\boldsymbol{P_{chr}}$, $\boldsymbol{P_{conj}}$, or $\boldsymbol{P_{non\text{-}conj}}$ initiated at different ratios (1:100, 1:1 and 100:1) and grown with different imipenem (IMP) concentrations. Dots and lines show individual data points and the mean (n = 4), respectively. p_{adj} values were derived by one-way ANOVA with post-hoc Tukey test and shown as *: $0.01 < p_{adj} < 0.05$; **: $0.001 < p_{adj} < 0.01$; ***: $p_{adj} < 0.001$.

Table S1. Minimal inhibitory concentrations (MICs) of strains used in this study

Strains	Imipenem MIC (µg/ml)	Tetracycline MIC (µg/ml)
E. coli MG1655-*mcherry* (***N***)	0.5	6
E. coli MG1655-*sfGFP-bla$_{KPC-2}$* (***P_{chr}***)	>30	6
E. coli MG1655-*mTagBFP2*/pKJK5-*sfGFP-bla$_{KPC-2}$* (***P_{conj}***)	>30	>50
E. coli MG1655-*mTagBFP2*/pKJK5$_{NC}$-*sfGFP-bla$_{KPC-2}$* (***$P_{non-conj}$***)	>30	>50
E. coli MG1655/pKJK5-*sfGFP-bla$_{KPC-2}$* (***P_{conj}***)	>30	>50
E. coli MG1655/pKJK5-*mTagBFP2* (***$N_{conj\ n-coop}$***)	0.5	Not tested

Table S2. The performed statistical tests

The performed statistical tests are listed and the statistics *e.g.* mean, slope, t value, sample size, *p* value, adjusted *p* value (padj), etc. are shown. *p* values are obtained with analysis of variance (ANOVA), Welch's t-test, or linear regression and adjusted with either Tukey's test or the Benjamini-Hochberg method.

Table S3. Average cell numbers of sub-populations and global populations over six days

Strain	Ratio	Treatment	Type (cell number, %)			Sum of the averaged cell number
			N (plasmid-free)	*P*	*T*	*N* (plasmid-free)+*P*+*T*
P_{chr}	1:1	AB-free	0.70x10^10, 54.3%	0.59x10^10, 45.7%	-, -	1.28x10^10
P_{chr}	1:1	IMP	0.09x10^10, 8.1%	1.03x10^10, 91.9%	-, -	1.12x10^10
P_{chr}	1:100	AB-free	1.27x10^10, 96.5%	0.05x10^10, 3.5%	-, -	1.32x10^10
P_{chr}	1:100	IMP	0.05x10^10, 4.4%	1.00x10^10, 95.6%	-, -	1.05x10^10
P_{chr}	100:1	AB-free	0.01x10^10, 1.1%	1.16x10^10, 98.9%	-, -	1.18x10^10
P_{chr}	100:1	IMP	0.00x10^10, 0.4%	1.15x10^10, 99.6%	-, -	1.15x10^10
P_{chr}	**Total**	AB-free	1.98x10^10, 52.5%	1.80x10^10, 47.5%	-, -	3.78x10^10
P_{chr}	**Total**	IMP	0.14x10^10, 4.3%	3.19x10^10, 95.7%	-, -	3.33x10^10
P_{conj}	1:1	AB-free	0.52x10^10, 40.8%	0.26x10^10, 20.8%	0.49x10^10, 38.4%	1.26x10^10
P_{conj}	1:1	IMP	0.03x10^10, 2.3%	0.95x10^10, 84.3%	0.15x10^10, 13.4%	1.13x10^10
P_{conj}	1:100	AB-free	1.20x10^10, 85.6%	0.01x10^10, 1.0%	0.19x10^10, 13.4%	1.40x10^10
P_{conj}	1:100	IMP	0.04x10^10, 4.0%	0.86x10^10, 84.9%	0.11x10^10, 11.1%	1.02x10^10
P_{conj}	100:1	AB-free	0.00x10^10, 0.0%	1.16x10^10, 98.0%	0.02x10^10, 2.0%	1.18x10^10
P_{conj}	100:1	IMP	0.00x10^10, 0.0%	1.00x10^10, 99.1%	0.01x10^10, 0.9%	1.01x10^10
P_{conj}	**Total**	AB-free	1.72x10^10, 44.6%	1.43x10^10, 37.3%	0.70x10^10, 18.1%	3.85x10^10
P_{conj}	**Total**	IMP	0.07x10^10, 2.1%	2.81x10^10, 89.2%	0.27x10^10, 8.7%	3.15x10^10
$P_{non-conj}$	1:1	AB-free	1.17x10^10, 88.4%	0.15x10^10, 11.6%	-, -	1.33x10^10
$P_{non-conj}$	1:1	IMP	0.41x10^10, 48.8%	0.43x10^10, 51.2%	-, -	0.84x10^10
$P_{non-conj}$	1:100	AB-free	1.46x10^10, 98.6%	0.02x10^10, 1.4%	-, -	1.48x10^10
$P_{non-conj}$	1:100	IMP	0.14x10^10, 18.0%	0.63x10^10, 82.0%	-, -	0.76x10^10
$P_{non-conj}$	100:1	AB-free	0.11x10^10, 11.0%	0.87x10^10, 89.0%	-, -	0.98x10^10
$P_{non-conj}$	100:1	IMP	0.03x10^10, 2.8%	0.89x10^10, 97.2%	-, -	0.91x10^10
$P_{non-conj}$	**Total**	AB-free	2.74x10^10, 72.5%	1.04x10^10, 27.5%	-, -	3.79x10^10
$P_{non-conj}$	**Total**	IMP	0.57x10^10, 22.7%	1.94x10^10, 77.3%	-, -	2.51x10^10

"AB-free": Without antibiotics; "IMP": 0.6 μg/ml imipenem; "***N***": Non-producers; "***P***": β-lactamase producres;

"***T***": Transconjugants.

Table S4. Average cell numbers in global populations

Table S5. Strains and plasmids used in this study

Strain name	Plasmids	Relevant genotype	Re-sistance	Reference
E. coli MG1655	-	Wide type	-	(9)
E. coli MG1655-*mcherry* (***N***)	-	$P_{A1\text{-}O4/O3}$-*mcherry*	KAN	This study
E. coli MG1655-*sfGFP-KPC2* (***P**chr*)	-	$P_{A1\text{-}O4/O3}$-*sfGFP-KPC2*	IMP	This study
E. coli/pGRG36	pGRG36	*Tn7*	AMP	(10)
E. coli/pGRG36-*mcherry*	pGRG36-P_{lpp}-*mcherry*	*Tn7*, P_{lpp}-*mcherry*	AMP, KAN	(6)
E. coli/pGRG36-P_{lpp}-*mTagBFP2*	pGRG36-P_{lpp}-*mTagBFP2*	*Tn7*, P_{lpp}-*mTagBFP2*	AMP, KAN	This study
E. coli/pGRG36-$P_{A1\text{-}O4/O3}$-*mTagBFP2*	pGRG36-$P_{A1\text{-}O4/O3}$-*mTagBFP2*	*Tn7*, $P_{A1\text{-}O4/O3}$-*mTagBFP2*	AMP, KAN	This study
E. coli/pKD46	pKD46	λ-Red recombination system	AMP	(3)
E. coli/pFLP2	pFLP2	FLP	AMP	(2)
E. coli/mTagBFP2-pBAD	mTagBFP2-pBAD	mTagBFP2	AMP	(5)
E. coli/pKJK5-*att*Tn7	pKJK5-*att*Tn7	IncP-1	TET	(4)
E. coli/pKJK5$_{NC}$-*att*Tn7	pKJK5 $_{NC}$-*att*Tn7	IncP-1	TET	(4)
E. coli/pGRG36-*sfGFP-KPC2*	pGRG36-*sfGFP-KPC2*	$P_{A1\text{-}O4/O3}$-*sfGFP-KPC2*	AMP, IMP	This study
E. coli MG1655-*mTagBFP2*/pKJK5-*sfGFP-KPC2* ($\boldsymbol{P_{conj}}$)	pKJK5-*sfGFP-KPC2*	$P_{A1\text{-}O4/O3}$-*sfGFP-KPC2*	TET, IMP	This study
E. coli MG1655-*mTagBFP2*/pKJK5$_{NC}$-*sfGFP-KPC2* ($\boldsymbol{P_{non\text{-}conj}}$)	pKJK5 $_{NC}$-*sfGFP-KPC2*	$P_{A1\text{-}O4/O3}$-*sfGFP-KPC2*	TET, IMP	This study
E. coli MG1655/pKJK5-*sfGFP-KPC2* ($\boldsymbol{P_{conj}}$)	pKJK5-*sfGFP-KPC2*	$P_{A1\text{-}O4/O3}$-*sfGFP-KPC2*	TET, IMP	This study (co-culture with $\boldsymbol{N_{conj\ n\text{-}coop}}$ and ***N***)
E. coli MG1655/pKJK5-*mTagBFP2* ($\boldsymbol{N_{conj\ n\text{-}coop}}$)	pKJK5-$P_{A1\text{-}O4/O3}$-*mTagBFP2*	$P_{A1\text{-}O4/O3}$-*mTagBFP2*	TET	This study (co-culture with $\boldsymbol{P_{conj}}$ and ***N***)

Table S6. Primers used throughout this study

Primer	Sequence 5'→3'
*att*Tn7Gen^{R}-F	GGGTGGATCTCGTTGTGGAT
*att*Tn7Gen^{R}-R	CCAAGATCGGATAACAGCACG
KPC2-F	ACTTCGGAATAGGAACTTCGACATCGCCTTGGGTTAC
KPC2-R	CGAAGCGGGTTTTTACGTAGACTTACTGCCCGTTGAC
pGRG36QQ-F	ATCAACGTATCAGTCGGGCG
pGRG36QQ-R	GCACTACTCAACCCCACGAT
PGRG36KanPlpp-1-F	CCTAGCAAACTGGGGCACAAGCTTAATTAAGTCGACGAATTAG-CCCGGG
PGRG36KanPlpp-1-R	AATGTAGCTAGCCTAACCACTCACTCTTCCCCATAAACCA
PGRG36KanPlpp-2-F	TGGTTTATGGGGAAGAGTGAGTGGTTAGGCTAGCTACATT
PGRG36KanPlpp-2-R	CTCCTTAATCAGCTCTTCGCCCTTAGACACCATATGTAATACCCTCTAG
bfp-F	GAGATTAACTCAATCTAGAGGGTATTACATATGGTGTCTAAGGGCG
bfp-R	GCCTTTCGCCCGGGCTAATTCGTCGACTTAATTAAGCTTGTGC
PGRG36-PA1-F	TCGAGATCGTCCGGGCCGCAAGCTCCTAGCGTTTTAATTAATCAG
PGRG36-PA1-R	CTCCTTAATCAGCTCTTCGCCCTTAGACACCATATGTAAATCCTCCTT
PGRG36PA10403-*bfp*-F	TTCACACAGCCTAATAAGGAGGATTTACATATGGTGTCTAAGGGCGA
PGRG36PA10403-*bfp*-R	AGGTGCTCGAGTGGCGGCCGCTATTGACCCTTAATTAAGCTTGTGC
PGRG36PA1-*bfp*-1-F	CTCCCTAGCAAACTGGGGCACAAGCTTAATTAAGGGTCAA-TAGCGGCC
PGRG36PA1-2-R	GAGCGAACGATCAAAAATAAGTGCCTTCCCGTGGCTTACTAG-GATCCG
PGRG36PA1-3-F	TTTAAATGATATCGGATCCTAGTAAGCCACGGGAAGGCACTTATT
PGRG36PA1-3-R	TGCAGCAAGCGGGATCTGATTAATTAAAACGCTAGGAGCTTGCGG

References

1. Green MR, Sambrook J. Molecular cloning: a laboratory manual. 4th ed. Cold Spring Harbor laboratory Press, NY, 2012.
2. Choi KH, Schweizer HP. mini-Tn7 insertion in bacteria with single attTn7 sites: example *Pseudomonas aeruginosa*. Nat Protoc. 2006;1:153–61.
3. Datsenko KA, Wanner BL. One-step inactivation of chromosomal genes in *Escherichia coli* K-12 using PCR products. Proc Natl Acad Sci U S A. 2000;97:6640–5.
4. Wang Q, Olesen AK, Maccario L, Madsen JS. An easily modifiable conjugative plasmid for studying horizontal gene transfer. Plasmid. 2022;123:102649.
5. Subach OM, Cranfill PJ, Davidson MW, Verkhusha V V. An enhanced monomeric blue fluorescent protein with the high chemical stability of the chromophore. Rao J, editor. PLoS One. 2011;6:e28674.
6. Olesen AK, Pinilla-Redondo R, Hansen MF, Russel J, Dechesne A, Smets BF, et al. IncHI1A plasmids potentially facilitate horizontal flow of antibiotic resistance genes to pathogens in microbial communities of urban residential sewage. Mol Ecol. 2022;31:1595–608.
7. Wiegand I, Hilpert K, Hancock REW. Agar and broth dilution methods to determine the minimal inhibitory concentration (MIC) of antimicrobial substances. Nat Protoc. 2008;3:163–75.
8. Sprouffske K, Wagner A. Growthcurver: an R package for obtaining interpretable metrics from microbial growth curves. BMC Bioinformatics. 2016;17:172.
9. Edwards JS, Palsson BO. The *Escherichia coli* MG1655 in silico metabolic genotype: Its definition, characteristics, and capabilities. Proc Natl Acad Sci. 2000;97:5528–33.
10. McKenzie GJ, Craig NL. Fast, easy and efficient: site-specific insertion of transgenes into enterobacterial chromosomes using Tn7 without need for selection of the insertion event. BMC Microbiol. 2006;6: 1–7.
