# Supplementary material for: Cooperative antibiotic resistance facilitates horizontal gene transfer
Source: ISME J. 2023 Mar 22;17(6):846–54. doi: 10.1038/s41396-023-01393-1 (PMC10203111; doi:10.1038/s41396-023-01393-1)
Supplement: Supplementary file 2 — Supplementary Table 2 [file 41396_2023_1393_MOESM2_ESM.docx]

**Table S2. The performed statistical tests.**

| **Condition** | **Group1** | **Group2** | **Group1_ mean** | **Group2_ mean** | ***p_adj_*** | **Test** | **Results** |  |
| --- | --- | --- | --- | --- | --- | --- | --- | --- |
| Supernatant | Pchr(n=9) | Pconj(n=9) | 0.381 | 0.600 | < 0.001 | ANOVA+ Tukey | Fig. 1A, mean |  |
| Supernatant | Pchr(n=9) | Pnon-conj(n=9) | 0.381 | 0.691 | < 0.001 |  |  |  |
| Supernatant | Pchr(n=9) | Pchr(n=9) | 0.381 | 0.011 | < 0.001 |  |  |  |
| Supernatant | Pconj(n=9) | Pnon-conj(n=9) | 0.600 | 0.691 | 0.297 |  |  |  |
| Supernatant | Pconj(n=9) | Pchr(n=9) | 0.600 | 0.011 | < 0.001 |  |  |  |
| Supernatant | Pnon-conj(n=9) | Pchr(n=9) | 0.691 | 0.011 | < 0.001 |  |  |  |
| Pellete | Pchr(n=9) | Pconj(n=9) | 0.965 | 1.241 | < 0.001 | ANOVA+ Tukey |  |  |
| Pellete | Pchr(n=9) | Pnon-conj(n=9) | 0.965 | 1.224 | < 0.001 |  |  |  |
| Pellete | Pchr(n=9) | Pchr(n=9) | 0.965 | 0.068 | < 0.001 |  |  |  |
| Pellete | Pconj(n=9) | Pnon-conj(n=9) | 1.241 | 1.224 | 0.823 |  |  |  |
| Pellete | Pconj(n=9) | Pchr(n=9) | 1.241 | 0.068 | < 0.001 |  |  |  |
| Pellete | Pnon-conj(n=9) | Pchr(n=9) | 1.224 | 0.068 | < 0.001 |  |  |  |
|  |  |  |  |  |  |  |  |  |
| **Condition** | **Group1** | **Group2** | **Group1_ mean** | **Group2_ mean** | ***p_adj_*** | **Test** | **Results** |  |
| Pconj-Pchr | Pconj(n=6) | Pchr(n=6) | 22.809 | 16.573 | 0.060 | ANOVA+ Tukey | Fig. 1E,  day1, mean |  |
| Pnon-conj-Pchr | Pnon-conj(n=6) | Pchr(n=6) | 38.934 | 16.573 | < 0.001 |  |  |  |
| Pnon-conj-Pconj | Pnon-conj(n=6) | Pconj(n=6) | 38.934 | 16.573 | < 0.001 |  |  |  |
|  |  |  |  |  |  |  |  |  |
| **Condition** | **Group1** | **Group2** | **Group1_ mean** | **Group2_ mean** | ***p_adj_*** | **Test** | **Results** |  |
| 1:1 IMP | Pchr(n=30) | Pconj(n=30) | 8.724 | 18.610 | 0.003 | linear regression+ emmeans+ Tukey | Fig. 1E,  day2-6, mean |  |
| 1:1 IMP | Pchr(n=30) | Pnon-conj(n=30) | 8.724 | 45.781 | < 0.001 |  |  |  |
| 1:1 IMP | Pconj(n=30) | Pnon-conj(n=30) | 18.610 | 45.781 | < 0.001 |  |  |  |
| 1:1 AB-free | Pchr(n=30) | Pconj(n=30) | 55.134 | 78.533 | < 0.001 | linear regression+ emmeans+ Tukey |  |  |
| 1:1 AB-free | Pchr(n=30) | Pnon-conj(n=30) | 55.134 | 89.859 | < 0.001 |  |  |  |
| 1:1 AB-free | Pconj(n=30) | Pnon-conj(n=30) | 78.533 | 89.859 | < 0.001 |  |  |  |
|  |  |  |  |  |  |  |  |  |
| **Condition** | **Group** | **Group_mean** | **t value** | ***p* value** | ***p_adj_*** | **Test** | **Results** |  |
| 1:1 day 1 | Pconj(n=6) | 22.8 | 8.083 | < 0.001 | < 0.001 | Welch’s t-test, one- sample  one-sided+ Benjamini-Hochberg | Fig. 2A,  IMP, mean |  |
| 1:1 day 1 | Pnon-conj(n=6) | 38.9 | 15.836 | < 0.001 | < 0.001 |  |  |  |
| 100:1 day 1 | Pconj(n=6) | 0.4 | 6.542 | < 0.001 | < 0.001 |  |  |  |
| 100:1 day 1 | Pnon-conj(n=6) | 1.6 | 6.604 | < 0.001 | < 0.001 |  |  |  |
| 1:100 day 1 | Pconj(n=6) | 40.5 | 13.422 | < 0.001 | < 0.001 |  |  |  |
| 1:100 day 1 | Pnon-conj(n=6) | 23.9 | 13.004 | < 0.001 | < 0.001 |  |  |  |
|  |  |  |  |  |  |  |  |  |
| **Condition** | **Group1** | **Group2** | **Group1_ mean** | **Group2_ mean** | ***p_adj_*** | **Test** | **Results** |  |
| 1:1 | N_rem_[Pconj](n=6) | N[Pchr](n=6) | 0.064 | 0.264 | 0.005 | ANOVA+ Tukey | Fig. 3A and Fig. S9, mean |  |
| 1:1 | N[Pnon_conj](n=6) | N[Pchr](n=6) | 0.584 | 0.264 | < 0.001 |  |  |  |
| 1:1 | T(n=6) | N[Pchr](n=6) | 0.358 | 0.264 | 0.372 |  |  |  |
| 1:1 | N[Pconj](n=6) | N[Pchr](n=6) | 0.332 | 0.264 | 0.677 |  |  |  |
| 1:1 | T(n=6) | N_rem_[Pconj](n=6) | 0.358 | 0.064 | < 0.001 |  |  |  |
| 1:1 | N[Pnon_conj](n=6) | N_rem_[Pconj](n=6) | 0.584 | 0.064 | < 0.001 |  |  |  |
| 1:1 | N[Pconj](n=6) | N_rem_[Pconj](n=6) | 0.332 | 0.064 | < 0.001 |  |  |  |
| 1:1 | N[Pconj](n=6) | N[Pnon_conj](n=6) | 0.332 | 0.584 | < 0.001 |  |  |  |
| 1:1 | T(n=6) | N[Pnon_conj](n=6) | 0.358 | 0.584 | 0.001 |  |  |  |
| 1:1 | N[Pconj](n=6) | T(n=6) | 0.332 | 0.358 | 0.985 |  |  |  |
| 1:100 | N_rem_[Pconj](n=6) | N[Pchr](n=6) | 0.215 | 0.136 | 0.849 | ANOVA+ Tukey |  |  |
| 1:100 | T(n=6) | N[Pchr](n=6) | 0.965 | 0.136 | < 0.001 |  |  |  |
| 1:100 | N[Pnon_conj](n=6) | N[Pchr](n=6) | 0.246 | 0.136 | 0.623 |  |  |  |
| 1:100 | N[Pconj](n=6) | N[Pchr](n=6) | 0.419 | 0.136 | 0.010 |  |  |  |
| 1:100 | N[Pnon_conj](n=6) | N_rem_[Pconj](n=6) | 0.246 | 0.215 | 0.994 |  |  |  |
| 1:100 | N[Pconj](n=6) | N_rem_[Pconj](n=6) | 0.419 | 0.215 | 0.095 |  |  |  |
| 1:100 | T(n=6) | N_rem_[Pconj](n=6) | 0.965 | 0.215 | < 0.001 |  |  |  |
| 1:100 | N[Pconj](n=6) | N[Pnon_conj](n=6) | 0.419 | 0.246 | 0.206 |  |  |  |
| 1:100 | T(n=6) | N[Pnon_conj](n=6) | 0.965 | 0.246 | < 0.001 |  |  |  |
| 1:100 | N[Pconj](n=6) | T(n=6) | 0.419 | 0.965 | < 0.001 |  |  |  |
| 100:1 | N[Pnon_conj](n=6) | N[Pchr](n=6) | 0.651 | 0.270 | < 0.001 | ANOVA+ Tukey |  |  |
| 100:1 | T(n=6) | N[Pchr](n=6) | 0.345 | 0.270 | 0.509 |  |  |  |
| 100:1 | N[Pconj](n=6) | N[Pchr](n=6) | 0.232 | 0.270 | 0.896 |  |  |  |
| 100:1 | T(n=6) | N[Pnon_conj](n=6) | 0.345 | 0.651 | < 0.001 |  |  |  |
| 100:1 | N[Pconj](n=6) | N[Pnon_conj](n=6) | 0.232 | 0.651 | < 0.001 |  |  |  |
| 100:1 | N[Pconj](n=6) | T(n=6) | 0.232 | 0.345 | 0.185 |  |  |  |
|  |  |  |  |  |  |  |  |  |
|  | **Group** | **slope** | **shappiro test  of residuals** | ***p* value** | ***p_adj_*** | **Test** | **Results** |  |
|  | N[Pchr](n=18) | 0.067 | 0.088 | 0.001 | 0.002 | linear  regression,  slope+ Benjamini-Hochberg | Fig. 3A and Fig. S9, slope |  |
|  | N_rem_[Pconj](n=18) | -0.151 | 0.321 | 0.004 | 0.008 |  |  |  |
|  | N[Pnon-conj](n=18) | 0.203 | 0.940 | 0.000 | < 0.001 |  |  |  |
|  | T(n=18) | -0.310 | 0.150 | 0.000 | < 0.001 |  |  |  |
|  | N[Pconj](n=18) | -0.094 | 0.128 | 0.007 | 0.008 |  |  |  |
|  |  |  |  |  |  |  |  |  |
| **Condition** | **Group1** | **Group2** | **Group1_ mean** | **Group2_ mean** | ***p_adj_*** | **Test** | **Results** |  |
| 1:1 | IMP(n=6) | AB_free(n=6) | 0.987 | 0.911 | < 0.001 | ANOVA+ Tukey | Fig. 3B, mean |  |
| 1:1 | TET(n=6) | AB_free(n=6) | 0.994 | 0.911 | < 0.001 |  |  |  |
| 1:1 | TET(n=6) | IMP(n=6) | 0.994 | 0.987 | 0.799 |  |  |  |
| 1:100 | IMP(n=6) | AB_free(n=6) | 0.641 | 0.285 | < 0.001 | ANOVA+ Tukey |  |  |
| 1:100 | TET(n=6) | AB_free(n=6) | 0.997 | 0.285 | < 0.001 |  |  |  |
| 1:100 | TET(n=6) | IMP(n=6) | 0.997 | 0.641 | < 0.001 |  |  |  |
| 100:1 | IMP(n=6) | AB_free(n=6) | 1.000 | 1.000 | 0.990 | ANOVA |  |  |
|  |  |  |  |  |  |  |  |  |
| **Condition** | **Group1** | **Group2** | **Group1_ mean** | **Group2_ mean** | ***p_adj_*** | **Test** | **Results** |  |
| 1:100 | TET(n=6) | IMP(n=6) | 0.109 | 0.524 | 0.130 | ANOVA+ Tukey | Fig. 3C, mean |  |
| 1:100 | AB-free(n=6) | IMP(n=6) | 1.641 | 0.524 | < 0.001 |  |  |  |
| 1:100 | AB-free(n=6) | TET(n=6) | 1.641 | 0.109 | < 0.001 |  |  |  |
| 1:1 | TET(n=6) | IMP(n=6) | 0.076 | 0.526 | < 0.001 | ANOVA+ Tukey |  |  |
| 1:1 | AB-free(n=6) | IMP(n=6) | 1.307 | 0.526 | < 0.001 |  |  |  |
| 1:1 | AB-free(n=6) | TET(n=6) | 1.307 | 0.076 | < 0.001 |  |  |  |
| 100:1 | AB-free(n=6) | IMP(n=6) | 0.145 | 0.065 | < 0.001 | ANOVA |  |  |
|  |  |  |  |  |  |  |  |  |
| **Condition** | **Group1** | **Group2** | **Group1_ mean** | **Group2_ mean** | **Days** | ***p_adj_*** | **Test** | **Results** |
| IMP | Pconj(n=6) | Pchr(n=6) | 6.8E+09 | 9.3E+09 | 1 | < 0.001 | annova+ Tukey | Fig. 4A, mean |
| IMP | Pconj(n=6) | Pchr(n=6) | 2.2E+10 | 2.6E+10 | 2 | < 0.001 |  |  |
| IMP | Pconj(n=6) | Pchr(n=6) | 4.8E+10 | 5.5E+10 | 3 | 0.003 |  |  |
| IMP | Pconj(n=6) | Pchr(n=6) | 8.1E+10 | 9.5E+10 | 4 | < 0.001 |  |  |
| IMP | Pconj(n=6) | Pchr(n=6) | 1.3E+11 | 1.5E+11 | 5 | < 0.001 |  |  |
| IMP | Pconj(n=6) | Pchr(n=6) | 1.9E+11 | 2.0E+11 | 6 | 0.128 |  |  |
| IMP | Pnon_conj(n=6) | Pchr(n=6) | 6.5E+09 | 9.3E+09 | 1 | < 0.001 |  |  |
| IMP | Pnon_conj(n=6) | Pchr(n=6) | 2.0E+10 | 2.6E+10 | 2 | < 0.001 |  |  |
| IMP | Pnon_conj(n=6) | Pchr(n=6) | 4.1E+10 | 5.5E+10 | 3 | < 0.001 |  |  |
| IMP | Pnon_conj(n=6) | Pchr(n=6) | 7.0E+10 | 9.5E+10 | 4 | < 0.001 |  |  |
| IMP | Pnon_conj(n=6) | Pchr(n=6) | 1.1E+11 | 1.5E+11 | 5 | < 0.001 |  |  |
| IMP | Pnon_conj(n=6) | Pchr(n=6) | 1.5E+11 | 2.0E+11 | 6 | < 0.001 |  |  |
| IMP | Pnon_conj(n=6) | Pconj(n=6) | 6.5E+09 | 6.8E+09 | 1 | 0.795 |  |  |
| IMP | Pnon_conj(n=6) | Pconj(n=6) | 2.0E+10 | 2.2E+10 | 2 | 0.140 |  |  |
| IMP | Pnon_conj(n=6) | Pconj(n=6) | 4.1E+10 | 4.8E+10 | 3 | < 0.001 |  |  |
| IMP | Pnon_conj(n=6) | Pconj(n=6) | 7.0E+10 | 8.1E+10 | 4 | < 0.001 |  |  |
| IMP | Pnon_conj(n=6) | Pconj(n=6) | 1.1E+11 | 1.3E+11 | 5 | < 0.001 |  |  |
| IMP | Pnon_conj(n=6) | Pconj(n=6) | 1.5E+11 | 1.9E+11 | 6 | < 0.001 |  |  |
| AB-free | Pconj(n=6) | Pchr(n=6) | 1.1E+10 | 1.1E+10 | 1 | 0.938 | annova+ Tukey |  |
| AB-free | Pconj(n=6) | Pchr(n=6) | 3.1E+10 | 2.9E+10 | 2 | 0.294 |  |  |
| AB-free | Pconj(n=6) | Pchr(n=6) | 6.3E+10 | 6.1E+10 | 3 | 0.606 |  |  |
| AB-free | Pconj(n=6) | Pchr(n=6) | 1.1E+11 | 1.1E+11 | 4 | 0.513 |  |  |
| AB-free | Pconj(n=6) | Pchr(n=6) | 1.6E+11 | 1.7E+11 | 5 | 0.144 |  |  |
| AB-free | Pconj(n=6) | Pchr(n=6) | 2.3E+11 | 2.3E+11 | 6 | 0.669 |  |  |
| AB-free | Pnon_conj(n=6) | Pchr(n=6) | 1.1E+10 | 1.1E+10 | 1 | 0.973 |  |  |
| AB-free | Pnon_conj(n=6) | Pchr(n=6) | 3.2E+10 | 2.9E+10 | 2 | 0.207 |  |  |
| AB-free | Pnon_conj(n=6) | Pchr(n=6) | 6.4E+10 | 6.1E+10 | 3 | 0.522 |  |  |
| AB-free | Pnon_conj(n=6) | Pchr(n=6) | 1.0E+11 | 1.1E+11 | 4 | 0.015 |  |  |
| AB-free | Pnon_conj(n=6) | Pchr(n=6) | 1.6E+11 | 1.7E+11 | 5 | 0.038 |  |  |
| AB-free | Pnon_conj(n=6) | Pchr(n=6) | 2.3E+11 | 2.3E+11 | 6 | 0.996 |  |  |
| AB-free | Pnon_conj(n=6) | Pconj(n=6) | 1.1E+10 | 1.1E+10 | 1 | 0.841 |  |  |
| AB-free | Pnon_conj(n=6) | Pconj(n=6) | 3.2E+10 | 3.1E+10 | 2 | 0.970 |  |  |
| AB-free | Pnon_conj(n=6) | Pconj(n=6) | 6.4E+10 | 6.3E+10 | 3 | 0.989 |  |  |
| AB-free | Pnon_conj(n=6) | Pconj(n=6) | 1.0E+11 | 1.1E+11 | 4 | 0.128 |  |  |
| AB-free | Pnon_conj(n=6) | Pconj(n=6) | 1.6E+11 | 1.6E+11 | 5 | 0.753 |  |  |
| AB-free | Pnon_conj(n=6) | Pconj(n=6) | 2.3E+11 | 2.3E+11 | 6 | 0.721 |  |  |
|  |  |  |  |  |  |  |  |  |
| **Condition** | **t value** | **slope** | **shappiro test  of residuals** | ***p* value** | **Test** | **Result** |  |  |
| non-cooperative  plasmid (n=60) | -14.92 | -2.2077 | 0.073 | < 0.001 | linear  regression | Fig. 5A, slope |  |  |
|  |  |  |  |  |  |  |  |  |
| **Condition** | **Group1** | **Group2** | **Group1_ mean** | **Group2_ mean** | ***p_adj_*** | **Test** | **Results** |  |
| AB-free | Non-cooperative  plasmid(n=180) | Cooperative  plasmid(n=180) | 53.5 | 42.9 | < 0.001 | linear  regression+  emmeans+ Tukey | Fig. 5A, day1-6, mean |  |
| AB-free | Non-cooperative  plasmid(n=180) | Plasmid free  cells(n=180) | 53.5 | 3.6 | < 0.001 |  |  |  |
| AB-free | Cooperative  plasmid(n=180) | Plasmid free  cells(n=180) | 42.9 | 3.6 | < 0.001 |  |  |  |
| IMP | Non-cooperative  plasmid(n=180) | Cooperative  plasmid(n=180) | 9.7 | 86.5 | < 0.001 | linear  regression+  emmeans+ Tukey |  |  |
| IMP | Non-cooperative  plasmid(n=180) | Plasmid free  cells(n=180) | 9.7 | 3.8 | < 0.001 |  |  |  |
| IMP | Cooperative  plasmid(n=180) | Plasmid free  cells(n=180) | 86.5 | 3.8 | < 0.001 |  |  |  |
|  |  |  |  |  |  |  |  |  |
| **Condition** | **Group1** | **Group2** | **Group1_ mean** | **Group2_ mean** | ***p_adj_*** | **Test** | **Results** |  |
| AB-free | Cooperative  plasmid(n=240) | Both(n=240) | 18.4 | 0.9 | < 0.001 | linear  regression+  emmeans+ Tukey | Fig. 5B, mean |  |
| AB-free | Non-cooperative  plasmid(n=240) | Both(n=240) | 21.5 | 0.9 | < 0.001 |  |  |  |
| AB-free | Both(n=240) | Cooperative  plasmid(n=240) | 0.9 | 18.4 | 0.974 |  |  |  |
| AB-free | Non-cooperative  plasmid(n=240) | None(n=240) | 21.5 | 3.6 | < 0.001 |  |  |  |
| AB-free | Non-cooperative  plasmid(n=240) | None(n=240) | 21.5 | 3.6 | < 0.001 |  |  |  |
| AB-free | Cooperative  plasmid(n=240) | None(n=240) | 18.4 | 3.6 | < 0.001 |  |  |  |
| IMP | Both(n=240) | Both(n=240) | 0.4 | 0.4 | 0.971 | linear  regression+  emmeans+ Tukey |  |  |
| IMP | Non-cooperative  plasmid(n=240) | Both(n=240) | 3.9 | 0.4 | < 0.001 |  |  |  |
| IMP | Non-cooperative  plasmid(n=240) | Cooperative  plasmid(n=240) | 3.9 | 28.2 | < 0.001 |  |  |  |
| IMP | Cooperative  plasmid(n=240) | None(n=240) | 28.2 | 3.8 | < 0.001 |  |  |  |
| IMP | Non-cooperative  plasmid(n=240) | None(n=240) | 3.9 | 3.8 | < 0.001 |  |  |  |
| IMP | Cooperative  plasmid(n=240) | None(n=240) | 28.2 | 3.8 | < 0.001 |  |  |  |
|  |  |  |  |  |  |  |  |  |
| **Condition** | **t value** | **Slope** | **shappiro test  of residuals** | ***p* value** | **Test** | **Result** |  |  |
| Non-cooperative  plasmid (n=36) | -8.053 | -2.0742 | 0.028 | < 0.001 | linear  regression | Fig. S11A, slope |  |  |

The performed statistical tests are listed and the statistics *e.g.* mean, slope, t value, sample size, *p* value, adjusted *p* value (*p_adj_*), etc. are shown. *p* values are obtained with analysis of variance (ANOVA), Welch’s t-test, or linear regression and adjusted with either Tukey’s test or the Benjamini-Hochberg method.
